# Extremely low thermal conductivity and high electrical conductivity of sustainable carbon­ceramic electrospun nonwoven materials

**DOI:** 10.1126/sciadv.ade6066

**Published:** 2023-03-31

**Authors:** Xiaojian Liao, Jakob Denk, Thomas Tran, Nobuyoshi Miyajima, Lothar Benker, Sabine Rosenfeldt, Stefan Schafföner, Markus Retsch, Andreas Greiner, Günter Motz, Seema Agarwal

**Affiliations:** ^1^ Macromolecular Chemistry 2 and Bavarian Polymer Institute, University of Bayreuth, Bayreuth 95440, Germany.; ^2^ Chair of Ceramic Materials Engineering, University of Bayreuth, Bayreuth 95440, Germany.; ^3^ Physical Chemistry 1 Department of Chemistry, Bavarian Polymer Institute, Bayreuth Center for Colloids and Interfaces, Bavarian Center for Battery Technology (BayBatt), University of Bayreuth, Bayreuth 95440, Germany.; ^4^ Bayerisches Geoinstitut, University of Bayreuth, Bayreuth 95440, Germany.; ^5^ Macromolecular Chemistry 2, Bavarian Polymer Institute, Bavarian Center for Battery Technology (BayBatt), University of Bayreuth, Bayreuth 95440, Germany.

## Abstract

Materials with an extremely low thermal and high electrical conductivity that are easy to process, foldable, and nonflammable are required for sustainable applications, notably in energy converters, miniaturized electronics, and high-temperature fuel cells. Given the inherent correlation between high thermal and high electrical conductivity, innovative design concepts that decouple phonon and electron transport are necessary. We achieved this unique combination of thermal conductivity 19.8 ± 7.8 mW/m/K (cross-plane) and 31.8 ± 11.8 mW/m/K (in-plane); electrical conductivity 4.2 S/cm in-plane in electrospun nonwovens comprising carbon as the matrix and silicon-based ceramics as nano-sized inclusions with a sea-island nanostructure. The carbon phase modulates electronic transport for high electrical conductivity, and the ceramic phase induces phonon scattering for low thermal conductivity by excessive boundary scattering. Our strategy can be used to fabricate the unique nonwoven materials for real-world applications and will inspire the design of materials made from carbon and ceramic.

## Teaser

Carbon/ceramic nonwovens with sea-island nanostructure perfectly balance extremely low thermal and high electrical conductivity.

## INTRODUCTION

Flexible, thermally stable, and flame-resistant nonwovens with tailored low thermal conductivity and high electrical conductivity are desirable materials (*1*, *2*). Because high thermal conductivity is proportional to high electrical conductivity, special strategies are required to invert the proportionality (*3*). Multiple strategies, like introducing strong anharmonicity (*4*), crystal complexity (*5*), heavy elements (*6*), clusters (*7*), entropy engineering (*8*), boundaries (*9*), size, and interface effects (*10*) are known for the achievement of a combination of low thermal conductivity ( about 50-1000 mW/m/K) and high electrical conductivity in the materials, like dense inorganic materials, conjugated polymers, and alloys. Some low-density porous carbon materials are known to exhibit low thermal conductivity and high electrical conductivity, for example, carbon-graphene composite aerogels (*11*) show electrical conductivity of 2.25 S/cm and thermal conductivity of 27 mW/m/K. Otherwise, the high density and conductive graphene films/fibers (*12*–*14*), carbon nanofiber nonwovens (*15*, *16*), and amorphous carbon (*17*) show high thermal conductivity (more than 10^4^ mW/m/K). Meanwhile, thermal stability is another practical challenge. While carbons have excellent stability in inert environments, they degrade at around 400°C in air (*18*). Recently, extremely low thermal conductivity (lower than air) could be achieved in flexible ceramic porous materials, like silica aerogels (15.9 mW/m/K) (*19*), hexagonal boron nitride aerogels (20 mW/m/K) (*20*), SiC@SiO_2_ nanowire aerogel (14 mW/m/K) (*21*), and Si_3_N_4_ nanofelts (11 mW/m/K) (Ar atmosphere) (*22*). Because of the inherent thermal stability and extremely low thermal conductivity, these ceramic materials show excellent flame resistance and high working temperature up to 1000°C. However, because of the correlation of low thermal conductivity and low electrical conductivity, such excellent thermal insulating materials are electrically isolating, which limits their applications in high technology, like electronics, energy, etc. Thus, achieving extremely low thermal conductivity in combination with high electrical conductivity is still a major challenge for flexible materials.

**Fig. 1.**
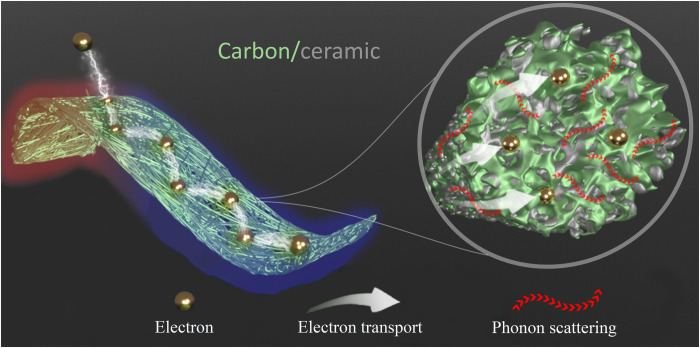
A schematic of the geometry of flexible carbon-ceramic composite fibrous nonwoven with the sea-island–type structure. The carbon phase modulates electronic transport, and the ceramic phase induces phonon scattering by excessive boundary scattering.

We found a facile concept for the combination of extremely low thermal conductivity and high electrical conductivity together with foldability and excellent fire resistance, as shown by the schematic illustration of electrospun carbon/silicon-based ceramic nanocomposite nonwoven materials (Fig. 1). The key for the discovery of the concept was the combination of carbon with nano-sized silicon-based ceramic inclusions in the form of a sea-island–type nanostructure (designated as C/SiCON) in the individual fibers (Fig. 1), derived from hybrid polymer materials: commercial polyacrylonitrile copolymer (PAN) and oligosilazane (OSZ) precursor (fig. S1) and the appropriate processing conditions. While PAN is used as a standard precursor for the carbon phase, the OSZ contributes to the formation of the nano-sized ceramic phase that is homogeneously distributed alongside the carbon phase in every single fiber in the form of a sea-island structure. We postulate that the increased density of interfaces between dissimilar materials (carbon and silicon-based ceramic), small pore size, and randomly laid solid fiber network structure result in the combination of extremely low thermal conductivity and high electrical conductivity.

## RESULTS

**Fig. 2.**
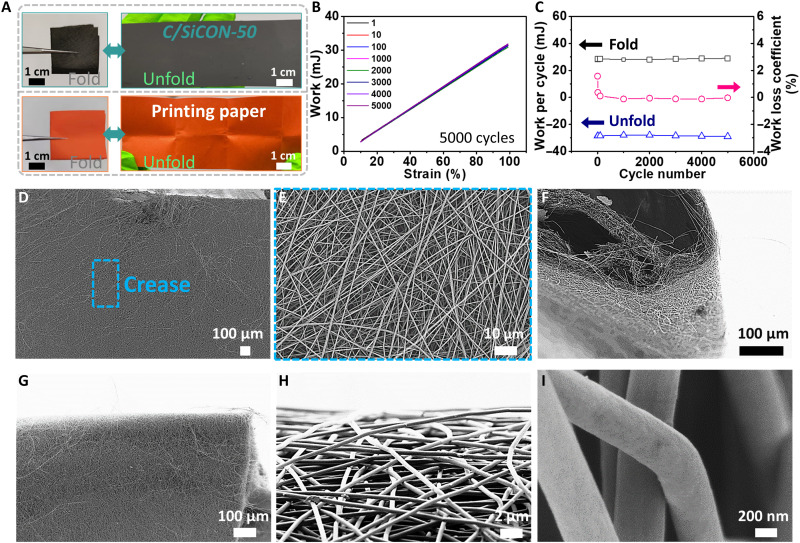
Robust and foldable C/SiCON-50 nonwovensABCD I (**A**) Digital images of C/SiCON-50 nonwoven showing the foldability of the material. The nonwoven can maintain its structure after being folded. The normal printing paper was used as a contrast test. (**B**) Work versus fold strain during a 5000-cycle folding-unfolding test with folding strain from 10 to 99%. (**C**) Changes of work and work loss coefficient per cycle with different cycle numbers during the 5000-cycle fold-unfolding test. (**D** to **I**) SEM images at three different angles of C/SiCON-50 nonwovens after the 5000-cycle fold-unfolding test in different magnifications. Top view of the surface images of unfolded nonwovens (D and E). It shows that a negligible amount of fibers in the membrane is broken. Side view (F) and front view (G) of flexible C/SiCON-50 nonwovens in a folded state. Images of deformed surfaces in high magnification (H and I).

The nonwovens were produced in three steps. At first, PAN/OSZ composite nonwovens were obtained by electrospinning of PAN and different amounts of OSZ. The nonwovens obtained by electrospinning were then stabilized by a step-wise temperature program or directly heating (fig. S2, A and B), from 20° to 250°C under the air atmosphere. Last, the stabilized nonwovens were carbonized and ceramized in an inert nitrogen atmosphere at 1000°C for 1 hour (fig. S2C), yielding the foldable C/SiCON composite nonwovens comparable to the printing paper (Fig. 2A). The polymer nonwovens are designated as PAN/OSZ-X, and the resulting carbonized and ceramized nonwovens are designated as C/SiCON-X, where X stands for the weight % (wt %) of OSZ relative to PAN in the electrospinning solution. Amounts ranging from 0 to 50 wt % of OSZ were investigated.

We found that the PAN fibers without OSZ had a near-circular shape with no beads and an average diameter of 900 ± 70 nm after electrospinning (fig. S3, A to C). The fiber shape and the diameter were retained after stabilization (figs. S3, A to F, and S4, A to C). The stabilization of PAN fibers by direct heating from 20° to 250°C at a heating rate of 2 K/min (fig. S2B), followed by carbonization resulted in brittle fibers. Insufficient hardening of the fibers caused melting and sticking of the fibers during carbonization, which led to a molten and brittle fibrous microstructure (fig. S5). We found that the stabilization with a step-wise temperature program from 20° to 250°C used to stabilize PAN fibers in this work was essential to avoid the melting of PAN and the fracturing of the fibers. Thus, after the carbonization of the stabilized nonwovens at 1000°C (N_2_; fig. S2C), flexible carbon nonwovens were obtained. The carbonization of fibers caused a reduction of the fiber diameter down to 470 ± 50 nm (fig. S6) because of an increase in density and mass loss. Raman spectra show typical carbon material peaks such as the G-line (a primary in-plane vibrational mode) and the disorder-induced D-line (fig. S7). Meanwhile, XRD (x-ray diffraction) was used to investigate the crystalline reflection of the carbon fibers. Typically, a broad peak at 2θ of approximately 25° is assigned to the disordered graphitic (0002) plane, and a weak peak at 2θ of about 43° belongs to the overlapping of the (10$\bar{1}$0) plane, and (10$\bar{1}$1) plane diffraction (fig. S7) (*23*). These broad peaks were the reflexes of ill-defined or small (nano-) crystallites.

The findings on PAN were transferred to the preparation of PAN/OSZ-X nonwovens. The fibers with an average diameter in the range of 500 to 600 nm were obtained using 10 to 30 wt % of OSZ in the electrospinning solution (fig. S6). The average fiber diameter increased to 1 to 1.6 μm when using higher amounts of OSZ (40 and 50 wt %) (figs. S6, S8, and S9). OSZs were reported to form advanced Si-based ceramics through a polymer˗to˗ceramic transformation at high temperatures (~1000°C) in an inert gas atmosphere (*24*). The carbonization and ceramization of the step-wise stabilized PAN/OSZ provided foldable and resilient C/SiCON nonwovens (see reversible foldability in Fig. 2A and movie S1) with an average fiber diameter ranging from 0.34 to 1.5 μm (fig. S6) and a porosity with a few micrometers in size (fig. S10). Compared to the obvious crease in the normal printing paper, we did not find the bold crease line in the C/SiCON nonwovens (Fig. 2A). In general, electrospinning has the advantage of modulating the mechanical properties based on the fiber diameter, which improves the foldability of the structure and can contribute to confining ceramic fillers (*25*, *26*). This versatility makes it a useful tool for the production of flexible hybrid materials with tailored properties. Furthermore, to test the materials’ fatigue resistance property, a 5000-cycle folding-unfolding test with compression strain from 10 to 99.0% was conducted (Fig. 2, B and C, and movie S2). A slight variation in work per cycle was found during this 5000-cycle folding-unfolding process, indicating that these C/SiCON-50 nonwovens have good fatigue tolerance, sufficient mechanical resilience, and foldability (Fig. 2B). The main changes in the work loss coefficient were very low and occurred in the first 10 cycles (from 1.6 down to 0.4%) (Fig. 2C). Meanwhile, after the 5000-cycle folding-unfolding test, the scanning electron microscopy (SEM) images in three perspectives (top, front, and side) revealed negligible damage to the fibers in the crease portion and bendable fibers (Fig. 2, D to I) compared to the pristine nonwovens (fig. S8, G to I).

**Fig. 3.**
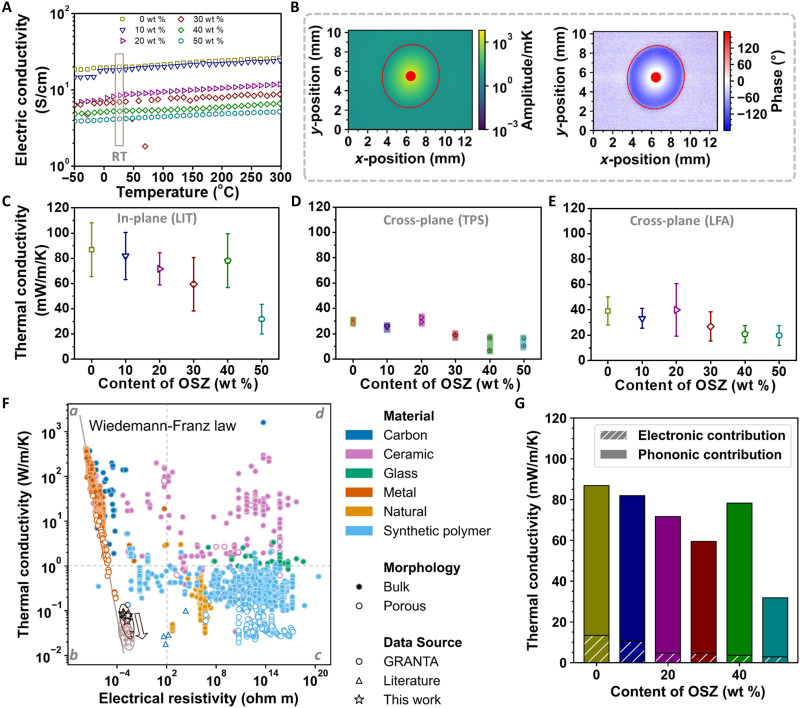
Thermal and electrical properties of the C/SiCON nonwovensABCD EFG (**A**) Temperature-dependent electrical conductivity of the C/SiCON-X nonwovens derived from PAN with different contents of OSZ. The measurements were conducted with the van der Pauw method. The marked area shows the RT electrical conductivity of the C/SiCON-X nonwovens versus the OSZ content. (**B**) LIT data for a C/SiCON-50 nonwoven. The excitation frequency is 0.5 Hz. After 40-s equilibration time, the sample was measured for 100 s. The resulting amplitude and phase were calculated using direct Fourier transformation. For the diffusivity evaluation, data along the two principal axes of the ellipse are taken into account. (**C**) In-plane thermal conductivity of the C/SiCON-X nonwovens derived from PAN with different contents of OSZ. Samples were measured with LIT. (**D** and **E**) Cross-plane thermal conductivity of the C/SiCON-X nonwovens derived from PAN with different contents of OSZ. (D) is measured via TPS, and (E) is measured via LFA. (**F**) Comparison of the thermal conductivity and electrical resistivity for C/SiCON-X nonwovens (in-plane thermal conductivity marked by black stars and cross-plane thermal conductivity marked by gray stars) and more than 3900 materials of all types in the Ansys Granta Selector database (www.grantadesign.com, date: 30 September 2021) and additional literature. The data of additional literature in this Ashby plot are shown in table S2. The arrow beside our work shows the decreasing trend of thermal conductivity as SiCON content increases. (**G**) Electronic and phononic contributions to the overall in-plane thermal conductivity of the C/SiCON-X nonwovens derived from PAN with different contents of OSZ. Samples were measured with LIT.

To determine the effect of the ceramic phase on the thermal and electrical properties of the nonwovens, the thermal conductivity and electrical conductivity of the C/SiCON nonwovens with varying SiCON contents were investigated. The increase of the SiCON ceramic phase decreased the electrical conductivity gradually from 20.1 to 4.2 S/cm at room temperature (RT) measured in the in-plane direction (Fig. 3A) but did not turn it into a complete insulator, indicating that the SiCON ceramic phase did not block electron transport through the conductive carbon phase in the fibers. A light-emitting diode (LED) lamp could be lighted using our flexible C/SiCON-50 nonwoven as an electric conductor. The brightness of the LED lamp visually did not change during bending, twisting, and folding operations (fig. S11 and movie S3). Meanwhile, the electrical resistance variation was recorded during the 5000-cycle fold-unfolding testing (movie S2). The C/SiCON-50 nonwovens revealed a negligible change in the electric conductivity after folding-unfolding 5000 times, represented by the electrical resistance variation (*R*/*R*_0_) of 1.01 (fig. S12). Moreover, we found that the electric conductivity increased slightly with increasing temperature from −50° to 300°C, which is known for semiconducting graphitic materials derived from pyrolyzed PAN and suggests our materials have a broad range of working temperatures (Fig. 3A). The thermal conductivity of the nonwoven was investigated along two distinct orientations: along (in-plane) and perpendicular (cross-plane) to the fiber orientation. We used lock-in thermography (LIT) for the in-plane transport, and light flash analysis (LFA) and transient plane source (TPS) characterization for the cross-plane transport (Fig. 3, B to E, and figs. S13 and S14). LIT and LFA determine the thermal diffusivity, from which the thermal conductivity is calculated by κ = α · ρ · *c*_P_, with κ being the thermal conductivity, α being the thermal diffusivity, ρ being the effective density, and *c*_P_ being the specific heat capacity. Increasing SiCON marginally raised the heat capacity, while the effective density of the nonwovens remained constant (about 100 mg/cm^3^) (figs. S13 and S14, A and B). The thermal diffusivity showed a clear falling trend with increasing SiCON content, particularly for the cross-plane thermal diffusivity (fig. S14, C and D). As a result, also the in-plane thermal conductivity decreased with increasing SiCON. We obtained the lowest in-plane thermal conductivity for C/SiCON-50 nonwovens (32 ± 12 mW/m/K), being well in the range of state-of-the-art polymer foams. The in-plane thermal transport measurements were performed in vacuum (<10^−2^ mbar). A similar decreasing trend of thermal conductivity was also observed in the cross-plane direction, which was all conducted under ambient conditions. First, the cross-plane thermal conductivity of C/SiCON-X nonwovens was measured via TPS. The minimum value was obtained for the C/SiCON-50 nonwovens with 10 ± 0.1 mW/m/K (in air, RT). Furthermore, LFA was used to confirm the cross-plane thermal conductivity. Although higher thermal conductivity values were recorded, like 19.8 ± 7.8 mW/m/K in the C/SiCON-50 nonwovens (in air, RT), it clearly showed the same trend as TPS: The higher content of SiCON, the lower cross-plane thermal conductivity. The lower thermal conductivity in the case of the TPS measurement can be attributed to an additional interfacial resistance between the measurement sensor and the nonwoven sample surface. Regardless, both cross-plane thermal conductivity values of C/SiCON-50 nonwovens are extremely low (thermal conductivity of air = 26 mW/m/K). Although the nonwovens constitute an open porous structure, heat transport via the gas phase apparently does not substantially add to the heat transport of these fibrous skeletons.

We highlight the unique combination of extremely low thermal conductivity and high electrical conductivity of our C/SiCON nonwovens by an Ashby plot (Fig. 3F) compared to more than 3900 materials of all types, including carbons, ceramics, natural materials, synthetic polymers, metals, glasses, and their composites in the formation of bulk or porous. Our C/SiCON nonwovens populate an empty space in quadrant b, where strong thermal insulation is combined with a low electric resistivity. This behavior could seemingly be expected when extrapolating the thermal conductivity and electrical conductivity of metals (orange) and carbonaceous (blue) materials; however, for ceramic materials, this property combination is quite remarkable. Moreover, our robust and foldable C/SiCON nonwovens are scalable as electrospinning is a continuous and industrialized production method for nonwovens.

Analyzing the electronic and phononic contributions to the overall thermal conductivity, we find that the electronic contribution is low and decreases with increasing OSZ content (Fig. 3G). Note that the exact electronic contribution strongly depends on the Lorenz number used for the calculation of the Wiedemann-Franz law. The Lorenz number has been reported to range between 2.45 × 10^−8^ and 4.60 × 10^−8^ WΩK^−2^ for graphitic materials (*27*). The calculation of the electronic contribution in Fig. 3G indicates the minimum electronic contribution.

**Fig. 4.**
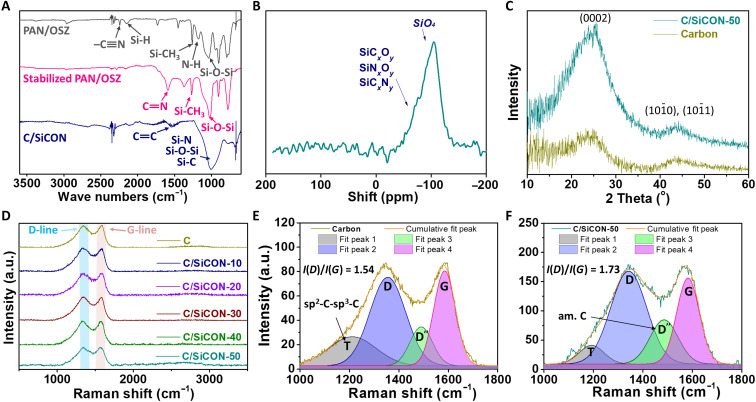
Analysis of the carbon and C/SiCON fibers. (**A**) ATR-FTIR spectra of the polymer, stabilized, and carbonized/ceramized nonwovens from PAN with 40 wt % OSZ. (**B**) Solid ^29^Si NMR spectra of C/SiCON-50 nonwovens. (**C**) XRD analysis of the pure carbon and C/SiCON-50 nonwovens. The (10$\bar{1}$0) plane, and (10$\bar{1}$1) plane reflexes overlap at 2θ near 43°. Table S3 shows the information on the nanocrystallites based on the XRD analysis. (**D**) Raman spectra of the pure carbon and C/SiCON nonwovens. (**E** and **F**) Curves fitted in Raman spectra of carbon fiber (E) and C/SiCON-50 (F) with a Gaussian function. The area values of (D) and (G) peaks were used to calculate the ratio of *I*(D)/*I*(G). a.u., arbitrary units.

**Fig. 5.**
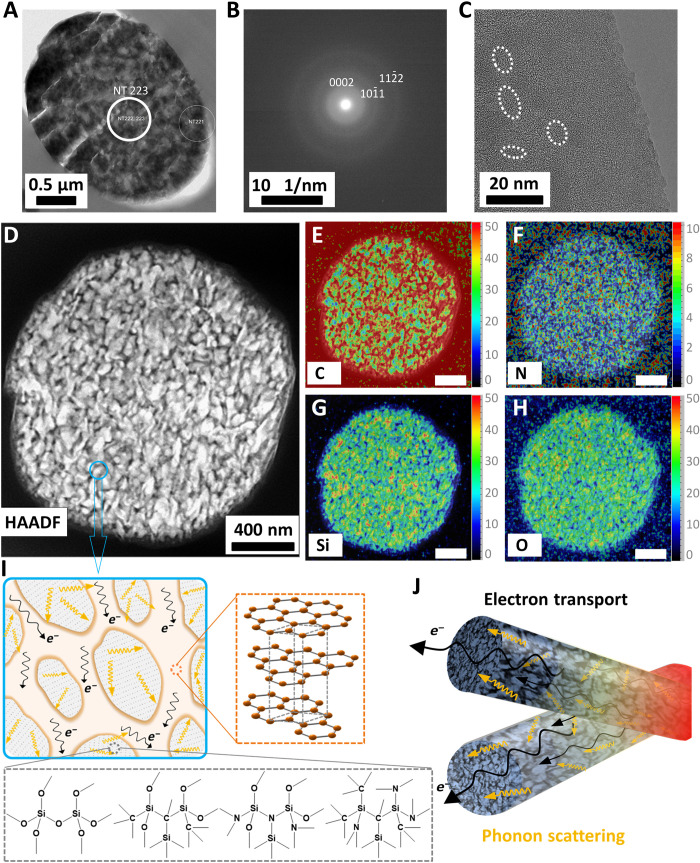
Nanostructure and elemental distribution of the C/SiCON fiber and the proposed model of thermal and electrical properties analysis. (**A**) TEM cross-sectional image of C/SiCON-50 fiber. (**B**) Selected area electron diffraction pattern (the position indicated the NT223 circle in the insert TEM cross-sectional image of C/SiCON-50 fiber) with three Debye rings originated from three strong (0002), (10$\bar{1}$1), and (11$\bar{2}$2) planes in the graphite-like structure (with sp^2^ and sp^3^ bonds). (**C**) High-resolution TEM micrographs of the cross section of the fiber. The marked areas show the basic structure of the nanocrystallites in the free carbon nanodomains. (**D** to **H**) High-resolution HAADF-STEM cross-sectional image (D) and EDS mapping (weight %) images (E to H) showing the cross section of a single C/SiCON-50 fiber. Note that the dominant components are C-bearing matrix (C-C or C-N) and Si-bearing inclusions (Si-O, Si-C, and Si-N) (dark and bright on HAADF-STEM image, respectively). Scale bars, 400 nm. (**I**) Schematic illustration of the sea-island nanostructure. (**J**) Schematic illustration of the electrical and thermal transport of C/SiCON fibers.

On the basis of our findings, we hypothesize that the sea-island–type structure, consisting of electrically conductive carbon as the matrix and a large number of phase boundaries owing to the dispersed thermally insulating SiCON inclusions (Fig. 1), resulted in nonwovens with the combination of extremely low thermal conductivity and high electrical conductivity. A pure carbon nonwoven alone is not suitable to reach an extremely low thermal conductivity rivaling the thermal insulation of air. ATR-FTIR (attenuated total reflectance–Fourier transform infrared spectroscopy) demonstrated overlapping signals of the Si-O-Si, Si-N, and Si-C (987 cm^−1^) belonging to the SiCON phase and the typical C═C (1500 cm^−1^) carbon phase (Fig. 4A and fig. S15). Evidently, during the solid ^29^Si-NMR [Si-29 nuclear magnetic resonance (NMR)] spectroscopy, a distinct SiO_4_ signal at −104 ppm (parts per million) was found. A broad peak in the −50 to −90 ppm region was characteristic for complex, mixed SiC*_x_*O*_y_*, SiN*_x_*O*_y_*, and SiC*_x_*N*_y_* environments (Fig. 4B). It indicated that the SiCON phase had been incorporated into the carbon phase. However, the addition of the SiCON phase did not result in substantial changes in XRD and Raman spectra, which we explain by overlapping broad peaks of the nano-crystaline reflexes of the carbon phase (Fig. 4, C to F). Compared to the pure carbon fibers, C/SiCON-50 fibers had a higher intensity ratio of the D band and G band [*I*(D)/*I*(G)] in the Raman spectra (Fig. 4, E and F), which indicates a higher disordering of the carbon phase and lower graphite content in the C/SiCON-50 fibers. The higher disordering carbon phase can complicate and extend the electron transport path and arouse additional scattering, which results in a decrease in the electrical conductivity and thermal conductivity. In addition, both d(0002) interplanar spacing and the size of nanocrystallites in *L*_a_ (crystallite lateral size) and *L*_c_ (crystallite thickness) of graphitic carbon in C/SiCON˗50 fiber were smaller than that of pure carbon fibers (table S3). Further investigation from the selected area electron diffraction pattern with three Debye-Scherrer rings in the cross-sectional TEM (transmission electron microscope) image of C/SiCON-50 fiber (Fig. 5, A to C) confirmed the graphitic (0002), (10$\bar{1}$1) planes observed in the XRD spectra, and additional (11$\bar{2}$2) and/or (11$\bar{2}$0) planes (d-spacing is about 0.12 nm, corresponding to the 2θ of approximately 78° in XRD spectra), while it also indicated a nano-crystalline structure in the C/SiCON-50 nonwovens. Furthermore, elemental analysis and XPS (x-ray photoelectron spectroscopy) investigated the elemental composition (table S4 and fig. S16). We observed that following stabilization and carbonization/ceramization of PAN/OSZ-50 nonwovens, the concentration of O and Si increased, whereas the quantity of N decreased, and the one of C remained constant (table S4). XPS surface survey of C/SiCON-50 fiber revealed an atomic ratio of Si species of 17.6 (equal to 29.4 wt %) (fig. S16), which is accompanied by a lower ratio of C species compared to the elemental analysis (table S4). Hence, a slight decrease of C at the surface of the fiber can be assumed for the C/SiCON-50 sample. Together, our analyses strongly indicate that carbon and SiCON were incorporated into the C/SiCON fibers by electrospinning, stabilization, and carbonization/ceramization. Furthermore, STEM-EDS (scanning transmission electron microscopy-energy-dispersive x-ray spectroscopy) spectroscopy was used to profoundly investigate the distribution and combination of elements inside the fibers (Fig. 5, D to H, and figs. S17 and S18). A high˗resolution HAADF (high-angle annular dark-field) cross section of a C/SiCON-50 fiber depicted bright blocks (heavy element, here Si) emerging from the dark or a gray area (light element, here C), similar to numerous islands distributed homogeneously in the sea (Fig. 5I) without the agglomeration of big blocks. The EDS mapping images show two dominant components: C-bearing matrix (C-C or C-N) and Si-bearing inclusions (Si-O, Si-C, and Si-N) (Fig. 5, D to H), consistent with the above results. We can see that the SiCON phase is distributed homogeneously within the carbon phase. This is also supported by the C/SiCON fibers with different contents of SiCON ceramic (C/SiCON-10 and C/SiCON-30; figs. S17 and S18), with a decrease in the sizes of the dispersed domains. All these results show that both carbon and SiCON phases are distributed homogeneously in the fiber with the formation of a sea-island nanostructure (*26*). On the basis of the two-dimensional slice through the fiber, we cannot discriminate between nanoparticulate inclusions inside the carbon matrix or the formation of a sponge-like co-continuous structure. In both cases, however, a high amount of interfaces can be expected inside the C/SiCON nanocomposite fibers. These interfaces, in combination with constriction resistances at the fiber-fiber contact points (fig. S9), lead to the unique combination of electric conductivity and thermal insulation.

The major achievement of this nanocomposite is that the increasing ceramic phase is not strongly increasing the electrical resistance and that the ceramic inclusions do not increase the phononic heat transport (Fig. 5). The uniformly dispersed SiCON phase introduces phonon boundary scattering within the carbon matrix (Fig. 5, I and J), resulting in a reduction in phononic thermal conductivity. This mechanism is also supported by the fact that a higher content of SiCON phase lowers the thermal conductivity (Fig. 3, C to F). Consequently, the C/SiCON nonwovens demonstrate optimum thermal insulation combined with an electrical conductivity close to the highest possible value as indicated by the extrapolated Wiedemann-Franz law (Fig. 3G). We attribute this unique combination to the intricate nanostructuring inside the C/SiCON fibers, where the presence of interfaces attenuates the phononic thermal transport of the ceramic parts, without impeding the electronic transport that is confined to the graphitic phase.

**Fig. 6.**
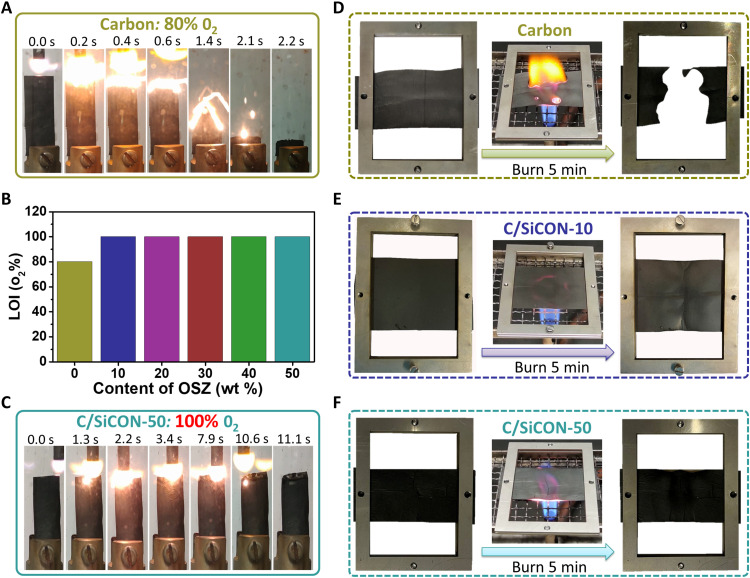
Flame resistant and thermal stability of C/SiCON nonwovens. (**A**) A set of real-time images showing the process of the LOI test on pure carbon nonwovens under 80% O_2_ atmosphere. (**B**) LOI values of the carbon nonwovens and C/SiCON nonwovens derived from PAN with different contents of OSZ. (**C**) A set of real-time images showing the process of the LOI test on C/SiCON-50 nonwovens under 100% O_2_ atmosphere. (**D** to **F**) Digital photos of carbon nonwovens (D), C/SiCON-10 nonwovens (E), and C/SiCON-50 nonwovens (F) before and after burning with a methane burner at about 800°C for 5 min in air.

Besides achieving the combination of extremely low thermal conductivity and high electrical conductivity, we also found that the C/SiCON nonwovens are nonflammable and thermally very stable (Fig. 6). The initial results showed that the pure carbon nonwoven could be easily ignited and burned out as the oxygen content is up to 80%, while the C/SiCON-50 nonwovens withstand and maintain the fiber form even at 100% O_2_ atmosphere in LOI (limiting oxygen index) tests (Fig. 6, A to C, and movie S4). As shown in the SEM images of both materials after LOI testing, the pure carbon fibers were burned into carbon dust with a block formation (fig. S19, A to D), while the C/SiCON-50 can remain in the fiber form with a melted state (fig. S19, E to H). Furthermore, the SEM-EDS surveys of both specimens were performed to investigate the element’s content (fig. S20). The carbon dust consists of carbon and oxygen with a weight ratio of 1:1. A clear increase of Si and O content (up to 43 and 46.5 wt %, respectively) and a decrease in C content (decrease to 10.4 wt %) were observed in the C/SiCON-50 nonwoven. On the basis of the above finding, we postulate that the homogeneously distributed SiOCN ceramic phase can remain and form a passivating silica layer on the surface after the surface carbon burn out, which can protect the fibers very effectively against further oxidation during the LOI testing. Our materials with outstanding fire resistance (LOI values of 100) are better than most polymers and carbon materials and are comparable to commercial ceramic materials, such as SiC, Al_2_O_3_, and SiO_2_ (fig. S21). In addition, a methane burner burning experiment in air (~800°C, 5 min) was conducted (Fig. 6, D to F, and movie S5). The carbon nonwovens were oxidized entirely, leaving no fiber residue (Fig. 6D). Whereas C/SiCON-10 and C/SiCON-50 nonwovens neither burnt nor showed any dripping and shape deformation (Fig. 6, E and F). Because the materials maintained their structural integrity, it makes them safer during fire hazards than pure carbon materials. After burning at 800°C for 5 min, the thermal conductivity and electrical conductivity of C/SiCON-50 nonwovens were also tested. The thermal conductivity remains at the extremely low value of about 16.6 mW/m/K (cross-plane), while the electrical conductivity drops to 0.02 S/cm due to a 36 wt % loss of carbon phase (fig. S22). This low electrical conductivity is still in the range of semiconductors. In the future, other concepts are required to maintain structural integrity during burning at higher temperatures and longer time while maintaining high electrical conductivity. This might require replacing carbon with other appropriate materials and making innovative phase-separated structures.

## DISCUSSION

A combination of extremely low thermal conductivity and high electrical conductivity was achieved by combining the SiCON ceramic phase and the carbon phase in electrospun fibers with a sea-island–type nanostructure. This enabled the simultaneous optimization of electron and phonon transport in the fibers. Thus, the electronic transport inside the carbon matrix phase could be retained without adding to thermal transport by phonon scattering at the SiCON ceramic interfaces. We are convinced that our underlying principle to combine dissimilar phases in the fibers to optimize phonon and electron transport is not just limited to the present system. The unique multifunctional properties of this textile-like material combine the best properties of various material classes, such as polymer-foam rivaling thermal insulation, ceramic-like fire retardance and nonflammability, and high electric conductivity. We are convinced that such materials with multifunctional properties would open up several application areas and tackle present bottlenecks for applications in the field of energy management, smart textiles, electromobility, or aerospace.

## MATERIALS AND METHODS

### Materials

PAN was obtained from Dolan GmbH (Germany) and used as received [copolymer with maximum 8% methyl acrylate and methallylsulfonate according to the datasheet from Dolan GmbH; number average molar mass (M*_n_*) = 95,000 g/mol; registration no. 26658-88-8]. OSZ Durazane 1800 was obtained from Merck (Germany) and used as received. Dicumylperoxide (DCP) was obtained from Sigma-Aldrich (Germany) and used as received. *N*, *N*′-dimethylformamide (DMF; 99.99%) and 99.9% acetone were obtained from Thermo Fisher Scientific GmbH (Germany) and used as received. The normal printing paper was Orange Label print paper from Canon, A4, 80 g/m^2^ (https://staples.de/orange-label-performance-papier-a4-80-g-m2-weiss/364513/).

### Electrospinning

The solution (~15 wt %) for electrospinning was prepared by dissolving PAN powder and Durazane 1800 (with 3 wt % of DCP) in DMF. After dissolving for 12 hours with a stirring speed of 500 rpm, acetone was added to the solution and stirred for another 2 hours at a speed of 500 rpm. The compositions of the solutions can be seen below in table S1.

The electrospun nonwovens were fabricated using a homemade setup with a syringe pump, a high-voltage direct-current power supply, and a rotary collector covered with aluminum foil. The solution was loaded into a syringe capped with a metal needle (diameter of 0.8 mm) connected with the high voltage. The polymer solution was pumped at a feed rate of about 0.8 ml/hour. The distance between the metal needle and collector was set at about 25 cm. After high voltage (+17 kV) was applied, polymer fibers were collected at the collector with a speed of 100 rpm and a diameter of about 10 cm for about 1.5 hours. The whole electrospinning process was conducted at RT and humidity of about 20%. Last, the polymer nonwovens were obtained after drying in a vacuum oven at 50°C for 48 hours.

### Stabilization process

The stabilization process was performed under air in a high-temperature clean room oven chamber furnace (Carbolite Gero, Germany). The polymer nonwovens were covered with graphite foil. A step-wise temperature program from 20° to 250°C with a heating rate of 2 K/min was used (fig. S2A). At 130°, 150°, 170°, 190°, 210°, 230°, and 250°C, the temperature was held for 1 hour each. The airflow rate was 4 liters/min. After cooling to RT by passive cooling within 1 day, the oxidized samples were obtained.

The control experiments were conducted by preparing carbon nonwovens. The PAN nonwovens were stabilized by heating from 20° to 250°C with a heating rate of 2 K/min and held at 250°C for 1 hour in the air (fig. S2B), and the airflow rate was 4 liters/min. After cooling down to RT by passive cooling within 1 day, the oxidized samples were obtained.

### Carbonization and ceramization process

The stabilized nonwovens were cut into strips with a length of 200 mm and a width of 50 mm. Each strip was covered with graphite foil and stacked layer by layer on the quartz glass board. The carbonization and ceramization process was performed in a FA100-500/13 tube oven furnace (Carbolite Gero, Germany) under a nitrogen atmosphere. The quartz glass board with samples was pushed into the heat zone position of tube oven from one side of the tube oven under the nitrogen atmosphere with N_2_ flow rate of 2 liters/min. After loading the samples and sealing the tube, the N_2_ flow rate was set to 150 ml/min. Then, samples were heated with different heating rates from 20° to 1000°C (fig. S2C): A slow heating rate of 1.25 K/min was used during RT to 100°C segment to flush out the oxygen contamination; a faster heating range with 2 K/min was used during 100° to 300°C segment and 700° to 1000°C segment; and during 300° to 700°C segment, a slower heating range of 1 K/min was set to ensure that the material has enough time for the carbonization and ceramization reactions to carbon and SiCNO ceramics. The holding time of 1 hour at 1000°C was used to complete the transformation of the material. Afterward, the furnace was cooled down to RT by passive cooling (without heating and natural cooling) within 1 day, the final materials in the quartz glass board were removed from the tube oven.

### Scanning electron microscopy

The SEM images of the nonwovens were acquired by the Zeiss LEO 1530 (Gemini, Germany) scanning electron microscope equipped with a field emission cathode and a secondary electron (SE2) and an Inlens detector. An acceleration voltage of 3 kV and a working distance between 5 and 6 mm were used. Before the measurement, the nonwoven samples were cut into small pieces and attached to a sample holder with conductive double-sided tape. The samples were subsequently sputter-coated with a 2.0-nm platinum layer by a Cressington 208HR high-resolution sputter coater, equipped with a quartz crystal microbalance thickness controller (MTM˗20). From 50-diameter measurements with the software ImageJ, an average value and the standard deviation of the fiber diameter were calculated.

### Folding-unfolding test

The 5000-cycle folding tests with compression strain from 10 to 99.0% were conducted to test materials’ fatigue resistance property. The sequential fold cycles were performed by the tensile tester (zwickiLine Z0.5, BT1-FR0.5TN.D14, Zwick/Roell, Germany) with a clamping length of 10 mm and a 20 N load cell (Zwick/Roell KAF TC). The samples were loaded between the two clamp stages with the top clamp stage applying uniaxial tension on the samples along the vertical direction. Meanwhile, a multimeter was connected to materials via copper conductors to record the electrical resistance during the folding test (movie S2). All curves were obtained at the strain ramp rate of 200 mm/min. The test time, cycle number, stress, stain, and the work per cycle were recorded. The work loss coefficient is calculated as the ratio of the work difference between the folding and unfolding of the each cycle to the folding work. After the 5000-cycle test, the samples were cut out from the specimen after suffering the 5000-cycle test with a scissor and used for SEM measurement. For the nonwoven sample’s side view of SEM, the nonwoven sample was maintained in a folded and vertical state in an SEM cross section using the accompanied screw. For the nonwoven sample’s front view of SEM, an unfolded nonwoven sample was attached to a conductive double-sided tape. All of the SEM measurements were carried out in the same way as the previous SEM measurements.

### ATR-FTIR spectroscopy

The ATR-FTIR studies were performed on a Tensor 27 system (Bruker, Germany) equipped with an ATR unit with a diamond crystal. After a background measurement, the nonwovens were cut into small pieces (about 5 mm by 5 mm) and pressed against the measuring diamond to receive a good signal. The measurements took place in a wave number range of 4000 to 400 cm^−1^ at a resolution of 5 cm^−1^. Thirty two measurements were averaged per sample to obtain higher signal-to-noise ratios. After the measurement, a baseline correction by the ATR-FTIR software (OPUS) was performed, and the measured data were saved as a .CSV file and plotted graphically using the Origin software.

### X-ray diffraction

XRD characterization was carried out using an anode x-ray generator (Bruker D8 ADVANCE, Karlsruhe, Germany) operating at 40 kV and 40 mA with Cu-*K*_α_ radiation (wavelength λ = 0.154 nm). Before the measurement, the nonwovens were fixed in a metal frame, and then the whole frame was installed in the instrument stage. XRD profiles were recorded in the 2θ angle range from 5° to 60° at a scanning speed of 0.05°/min at 25°C in transmission mode. The background was recorded by measuring an empty metal frame. The acquired XRD curves were analyzed by DIFFRAC.EVA V4.0 software, while the final spectra were obtained by background subtraction.

### Raman

A combined Raman Imaging/Scanning Force Microscope System (WITec alpha 300 RA+, Germany) with WiTec Control FIVE 5.3 software was used for RAMAN measurements. Laser is equipped with a UHTS 300 spectrometer combined with a back-illuminated Andor Newton 970 electron multiplying charge-coupled device camera [resolution: ca. 300 to 400 nm (lateral) and 900 nm (*z*) with 100× objective].

The measurements were carried out at an excitation wavelength of λ = 532 nm and a laser power of 1 mW with 50 accumulations with an integration time of 0.5 s pixel^−1^. The samples were stacked on a glass slide. After adjusting the focus on the nonwovens at ×100 magnification, the Raman spectrum was recorded. A cosmic ray removal and a baseline correction were performed on all spectra. The peaks were then fitted with a Gaussian function using the built in routine of Origin 2016.

### Thermal conductivity

Thermal conductivity was measured with three different methods: LIT, LFA, and TPS. Conductivity values during the LIT and LFA were calculated by κ = α · ρ · *c*_p_, with α being the thermal diffusivity, ρ being the effective density, and *c*_p_ being the specific heat capacity. The details are described in the following parts.

#### Thermal conductivity by TPS method

The thermal conductivity measurements were performed by the TPS method on a Hot Disk Thermal Constants Analyser (TPS 2500 S, thermal conductivity range: 0.005 to 1800 W/m/K, reproducibility: thermal conductivity ±3%, Hot Disk, Sweden) with a Kapton 7854 sensor operating with the thin-film module (thickness range: 20 to 600 μm) method at a constant temperature of 22°C controlled by air condition. The “Hot Disk Thermal Constants Analyser 7.4.15” software was used for the evaluation.

Before the measurement, two 30 mm by 30 mm squares were cut out of the sample. Each square’s thickness was measured with a digital micrometer at five points, corresponding to five cube eyes, and the average thickness was determined from the numerical mean value as the measurement parameter. The Kapton sensor (design 7854, radius = 10.5 mm) was placed centrally between the two square samples and placed in the measurement apparatus “slab sample holder” (Hot Disk Thermal Constants Analyser, Instruction Manual, Revision date 8 2019 October). The sensor in the sample sandwich was weighted with the upper parts of the slab sampler holder (a total mass of 304 g) and the additional stainless steel weights of 630 g (according to the stainless steel verification samples of the TPS 2500 S). The measuring thermometer with PT100 resistance was placed directly next to the measuring module, and the measuring arrangement was closed with the protective cover.

Before each series of measurements, the background thermal conductivity was measured once a day as a reference measurement. This reference measurement was carried out with a heating power of 750 mW and a measuring time of 20 s resulting in a thermal conductivity of 28.8 mW/m/K of the Kapton sensor and 14.9 W/m/K of the slab sample holder stack, respectively. After 1 hour, sample measurement was conducted five times with interval time at 1 hour. On the basis of the same background thermal conductivity, the sample measurements were carried out with heating power at 450 mW and measuring time at 20 s, so that all sample measurement results, including background thermal conductivity, background thermal diffusivity, background specific heat, probing depth, temperature increase, total to characteristic time, total temperature increase, and mean deviation, were within the parameter limits of the evaluation software.

#### Thermal diffusivity

Cross-plane thermal diffusivity was measured via LFA (Netzsch LFA 467 HT HyperFlash). Samples were cut in cylinders with 10 mm in diameter. Height was determined via laser microscopy as described below (see the “Effective density” section). Measurements were done under ambient conditions. The pulse width was set to 100 μs, lamp voltage to 230 V, and acquisition time to 50 ms. The data were fitted by a penetration model with Netzsch’ Proteus software. For each OSZ content, three samples were measured.

In-plane thermal diffusivity was measured via LIT using an improved version of our custom setup (*28*). Samples were cut in rectangles with a size of approximately 25 mm by 25 mm. They were put into a vacuum chamber (*P* < 10^−2^ mbar). A laser (Genesis MX 532-1000 SLM OPS, Coherent, λ=532 nm) periodically heated the samples at their center. The excitation frequency was varied between 0.1 and 1 Hz. After 20 drop periods, an IR camera (Image IR 9430, InfraTec GmbH) detected the temperature change for 100 s. With a frame rate of 20 fps, this resulted in 2000 images and a noise level of $\frac{30\;\mathrm{mK}}{\sqrt{2000}}<1\;\mathrm{mK}$. The amplitude and phase were calculated in real time using Infratec’s IRBIS active online software. The resulting data were evaluated with a custom Python package. It detects the excitation center and fits an ellipse to the amplitude. Afterward, all points on top of the two main axes of the ellipse are evaluated using the slope method (*29*). For each OSZ content, three samples were measured. The resulting diffusivity values for each sample at each frequency and the two different axes were averaged for the final in-plane diffusivity.

#### Effective density

The samples’ height for cross-plane thermal diffusivity characterization was measured via laser microscopy (LEXT OLS5000, Olympus IMS). Cylinders of 10 mm in diameter were cut from the nonwovens. Individual samples were put on the microscope stage. To ensure a flat contact, a glass slide was put on either side of the sample, leaving an unobstructed strip of about 5 mm in the center of the sample. A height image was acquired, and the average height of the sample was determined.

The mass of the samples was determined with a microscale (Cubis Micro Balance, Sartorius Lab Instruments GmbH). The volume was calculated from the known area (*A* = π*r*^2^ ≈ 78.5 mm^2^) and the measured height. The measurement was repeated for nine samples for each OSZ content. The given density is the average of all samples with the standard deviation being the error.

#### Heat capacity

Heat capacity was measured via differential scanning calorimetry (DSC; Discovery DSC 2500, TA instruments) of one sample for each OSZ content according to the American Society for Testing and Materials E1269-11 standard. All samples were ground to a powder before the measurement to ensure good contact with the DSC pans. Two heating cycles between −40° and 200°C were performed with a 20 K/min heating rate. Only the second heating cycle was evaluated. The measurement was repeated four times. The final value is the average of all measurement runs at 23°C.

### Temperature-dependent electric resistivity

Samples of about 25 mm by 25 mm size were measured via the van der Pauw method (*30*). Four spring-loaded probes were placed at the edges of the sample. A current of 0.1 mA was applied with a Keithley 6221 current source, while the voltage was simultaneously measured with a Keithley 6517 electrometer. The temperature of the sample was controlled with an Instec mK2000 controller. The resistance was measured from −50° to 300°C with a 2 K/min heating rate. Specific resistivity was calculated by measuring the thickness of the samples with a Mitutoyo Litematic VL-50.

### Nuclear magnetic resonance

NMR experiments were performed with a Bruker Avance II 300 (magnetic field of 7.05 T) spectrometer in a 4-mm triple resonance sample head (also from Bruker) at a rotation speed of 10 kHz. The ^29^Si MAS NMR measurements were performed using a single-pulse quantitative experiment with a 90° pulse length of 3.5 μs, a recycle delay of 60 s, and no proton decoupling during acquisition. The spectra are indirectly referenced with *N*(*SiMe*_3_)_3_/σ(*iso*) = 2.4 ppm, with respect to tetramethylsilanes [σ(*iso*) = 0.0 ppm].

### Pore size tests

Pore size measurements were carried out on a capillary flow porometer PSM 165/H (Dresden, Germany). The standard test liquid Topor (surface tension = 16.0 mN/m) was used. The specimens were cut into squares (length about 2 cm) and covered the sample holder hole. The sample holder diameter is 11 mm, and N_2_ flow rate was up to 70 liters/min.

### Elemental analysis

The measurements were carried out at Mikroanalytisches Labor Pascher (www.mikrolabor.com, Germany). According to the company, the following methods were used: carbon in the polymer, oxidized, and ceramic state.

The samples were burned with a combustion additive at about 1200°C in a stream of oxygen; the contained carbon burned to CO_2_. The CO_2_ was dissolved in sodium hydroxide solution, and the carbon content in the sample was calculated from the change in electrical conductivity (conductometry).

#### Hydrogen in the polymer, oxidized, and ceramic state

The samples were burned at 1050°C in an oxygen stream. The combustion water formed was determined by IR spectroscopy.

#### Nitrogen in the polymer and oxidized state

The sample was melted at a temperature of 900°C in the oxygen stream with a catalyst. Ammonia, N_2_, or NO*_x_* formed were passed over copper oxide and reduced to N_2_ after switching the carrier gas to CO_2_, excess oxygen was bound. The nitrogen formed was purged into an azotometer. Acidic reaction gasses and the carrier gas CO_2_ were bound by potassium hydroxide solution. The nitrogen gas was measured volumetrically.

#### Nitrogen in the ceramic state

The sample was degassed in a heated graphite crucible. The released nitrogen was measured with helium as carrier gas with thermal conductivity detection.

#### Oxygen in the polymer and oxidized state

Samples were pyrolyzed at 1500°C in a glass carbon tube with carbon contact. Oxygen was detected as CO and measured by thermal conductivity detection.

#### Oxygen in the ceramic state

The sample was degassed in a hot graphite crucible. The CO/CO_2_ produced by the reaction of the released oxygen with the graphite was pumped off and detected by IR spectroscopy.

#### Silicon in the polymer and oxidized state

After pressure digestion with nitric acid, the silicon dioxide formed was digested with sodium hydroxide solution under pressure. Detection was carried out by ICP-AES (inductively coupled plasma–atomic emission spectroscopy).

#### Silicon in the ceramic state

The sample was digested under melting (with a soda-borax mixture in a Pt crucible) and then dissolved with water. The detection was done by ICP-AES.

### Scanning transmission electron microscopy

We performed HAADF-STEM imaging and chemical analyses on a field emission scanning transmission electron microscope [Field Electron and Ion Company (FEI), Titan G2 80-200 S/TEM], equipped with an EDS (four silicon drift detectors, Bruker Quantax) and electron energy-loss spectrometer (EELS; Gatan Imaging Filter, Quantum SE) to observe and analyze the chemical compositions and relative thicknesses of the samples against a chemical standard of a C/SiCON nonwoven, respectively. For the TEM sample preparation, we used ultramicrotomy and Ar-milling methods to make thin cross-sectional specimens using an ultramicrotomy machine and Jeol Ion Slice. The TEM thin foils were a thickness of 50 to 150 nm, but we selected approximately the same thickness areas for the EDS by using zero-loss spectra in the EELS. The EDS maps were taken at a resolution of 5 to 10 nm per pixel and a dwell time of 16 μs using a subnanometer-sized electron beam with less than 0.09-nA probe current at 200-kV acceleration voltage. To accumulate statistically enough characteristic x-ray counts in a quantitative EDS map, the total acquisition time was about 60 min. During the acquisition, an image drift correction function was always activated to prevent artifacts in the profile. To get quantitative compositions of the samples, we corrected *z*-number and absorption effects on the evaluations of EDS spectrum, based on the chemical compositions of a C/SiCON-50 nonwoven measured in Laboratory Pascher.

### Limiting oxygen index

The LOI experiments were carried out on an Fire Testing Technology Oxygen Index Apparatus ISO 4589-3–NES 715 (Edenharter, Germany). The nonwoven samples were cut in shapes (width about 1 cm and length about 6 cm) and fixed in the device. The respective oxygen and nitrogen composition were adjusted before the measurement. Then, the samples were exposed to the oxygen and nitrogen composition atmosphere for 5 min and followed by burning with a methane flame. The composition of oxygen and nitrogen at which the sample burned down independently was recorded. When the methane flame was removed and even at 100% O_2_, the flame was immediately extinguished, and an LOI value of 100 O_2_% was recorded (movie S4).

### Burning tests

The burning test was conducted in a hood in the air atmosphere. For the burning tests, the nonwovens were cut in rectangular shapes (width: 4 cm and length: 6 cm) and fixed in a metal frame. The metal frame with the nonwoven was placed on a metal rack, with a methane burner installed beneath it. Then, the samples were burned with a methane flame (rate: 0.5 liter/min). After ignition, the samples were burned for 5 min, and the whole process was recorded by video (movie S5).

## Supplementary Materials

### This PDF file includes:

Figs. S1 to S22 Tables S1 to S4 Legends for movies S1 to S5 References
