# Supplementary material for: Extremely low thermal conductivity and high electrical conductivity of sustainable carbon­ceramic electrospun nonwoven materials
Source: Sci Adv. 2023 Mar 31;9(13):eade6066. doi: 10.1126/sciadv.ade6066 (PMC10065829; doi:10.1126/sciadv.ade6066)
Supplement: Supplementary file 1 — Figs. S1 to S22 Tables S1 to S4 Legends for movies S1 to S5 References [file sciadv.ade6066_sm.pdf]

Science Advances
AAAS

Supplementary Materials for

Extremely low thermal conductivity and high electrical conductivity of sustainable carbon-ceramic electrospun nonwoven materials

Xiaojian Liao *et al.*

Corresponding author: Seema Agarwal, agarwal@uni-bayreuth.de; Günter Motz, guenter.motz@uni-bayreuth.de; Markus Retsch, markus.retsch@uni-bayreuth.de

Sci. Adv. **9**, eade6066 (2023)
DOI: 10.1126/sciadv.ade6066

The PDF file includes:

Figs. S1 to S22
Tables S1 to S4
Legends for movies S1 to S5
References

Other Supplementary Material for this manuscript includes the following:

Movies S1 to S5

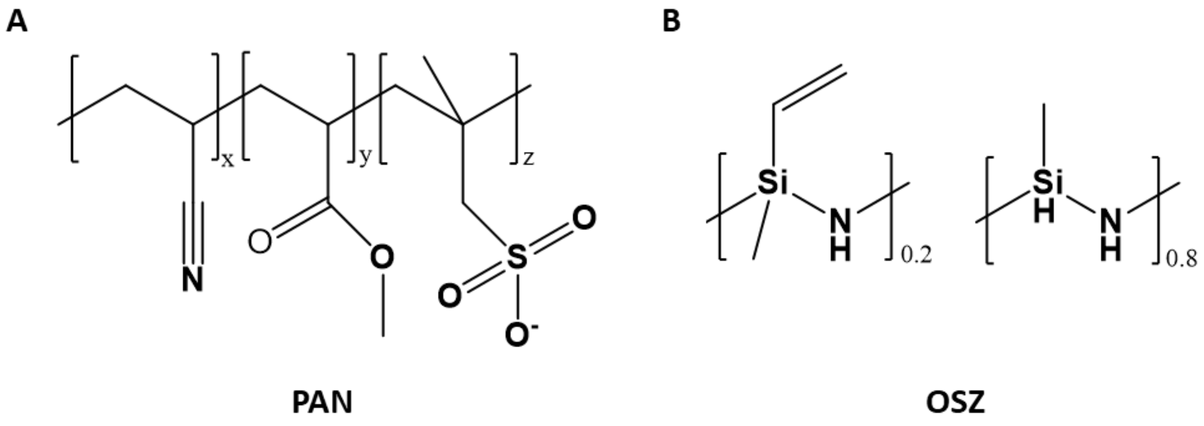

Fig. S1.

Chemical structures of the precursors. (**A**) polyacrylonitrile coplymer (PAN) and (**B**) oligosilazane (OSZ).

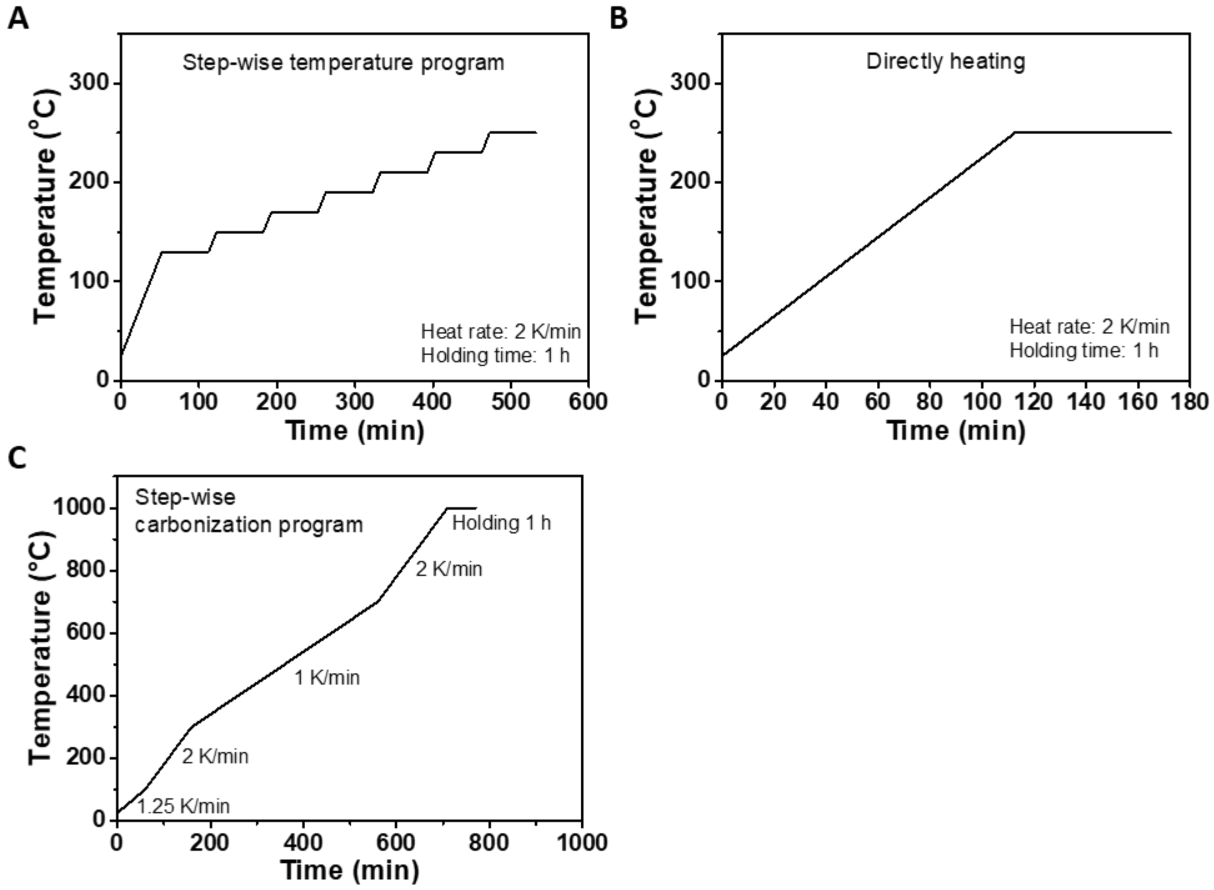

Fig. S2.

Heating program plots during the stabilization and the carbonization and ceramization processes. (**A**) Step-wise stabilization temperature program. (**B**) Directly stabilization heating program. (**C**) Step-wise carbonization and ceramization temperature program.

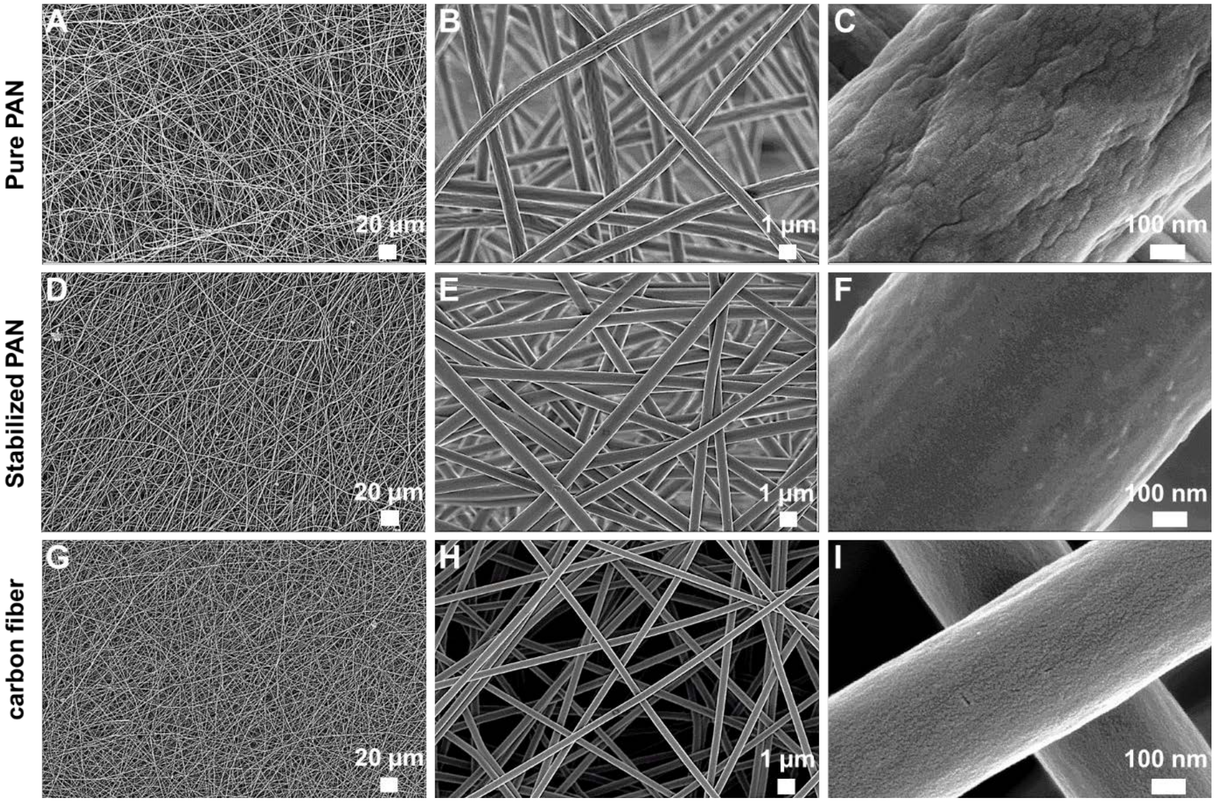

Fig. S3.

Surface SEM images of nonwovens with different magnifications. (**A-C**) Pure PAN. (**D-F**) Stabilized PAN nonwoven. (**G-I**) Carbon nonwoven.

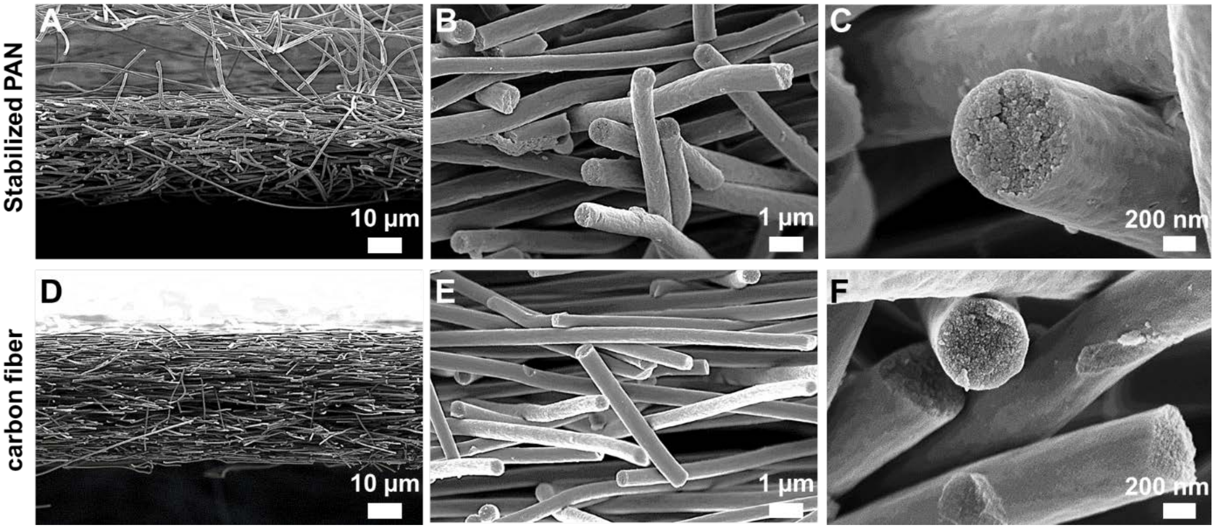

Fig. S4.

Cross-sectional SEM images of nonwovens with different magnifications. (**A-C**) stabilized PAN nonwoven. (**D-F**) carbon nonwoven.

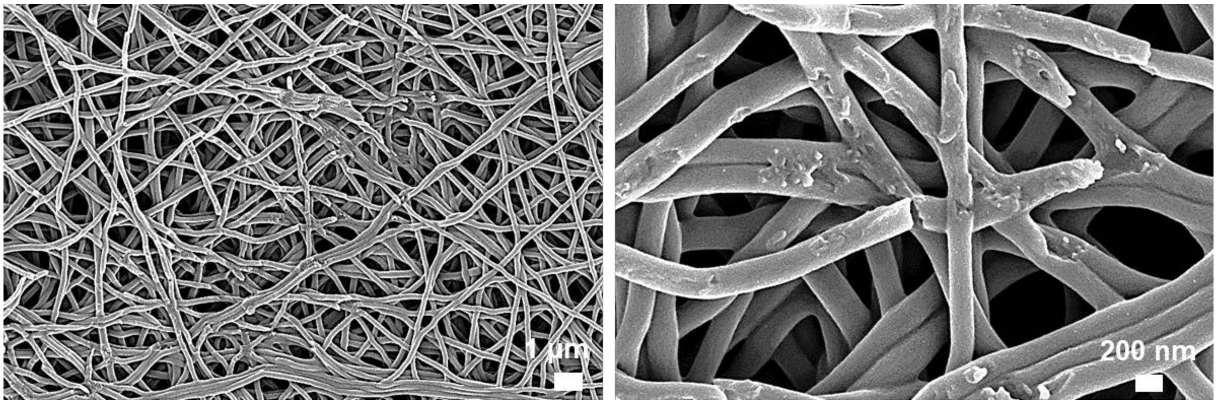

Fig. S5.

Surface SEM images of brittle pure carbon nonwoven in different magnifications. The samples were stabilized by heating from 20 °C to 250 °C with a heating rate of 2 K/min and holding the temperature at 250 °C for 1 h in the air. Afterward, the carbonization was performed under nitrogen in a tube oven, for which the samples were heated with 2 K/min to 1000 °C and annealed at this temperature for 1 h.

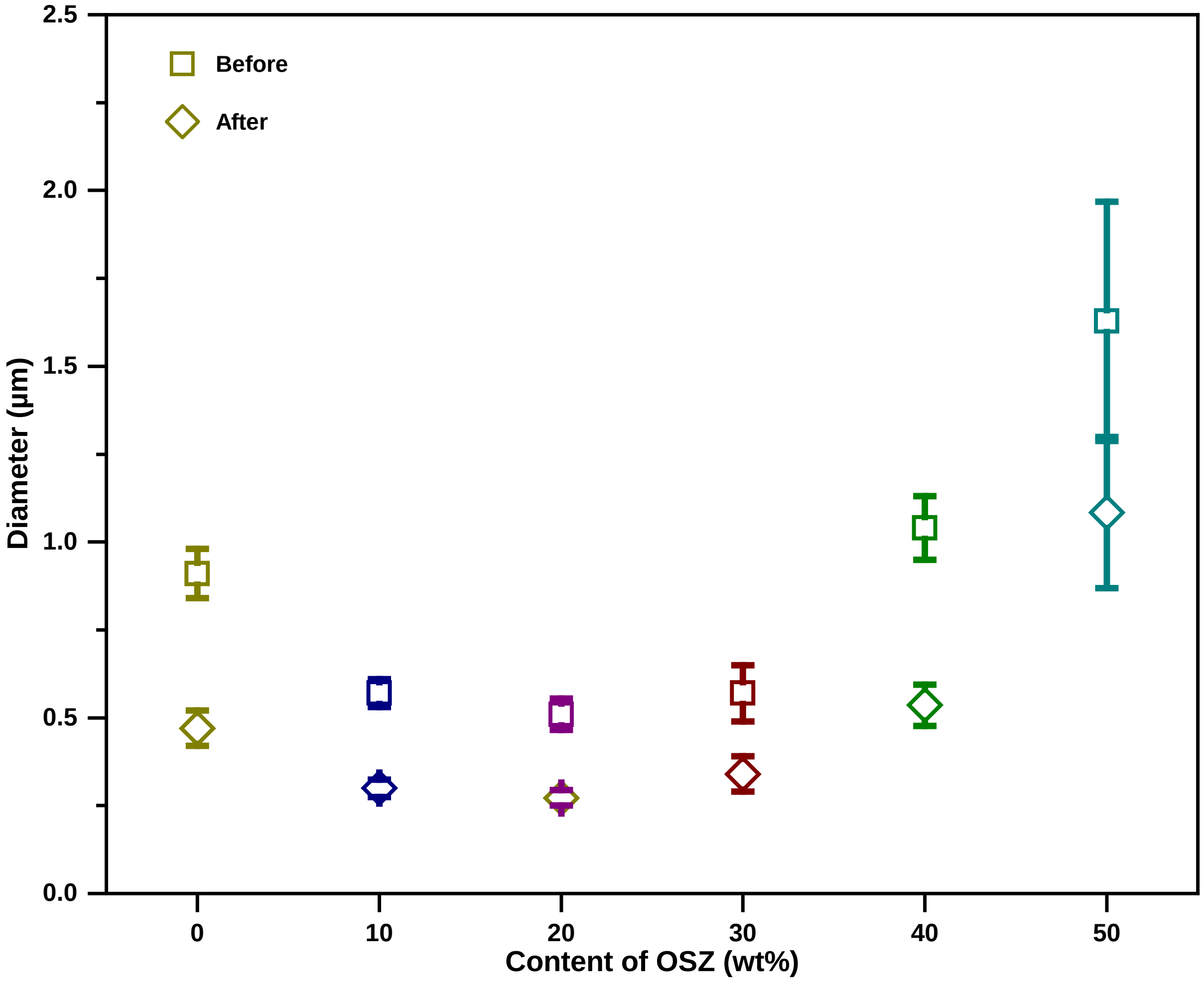

Fig. S6.

Changes in fiber diameter with the function of the content of OSZ. Before: polymer PAN/OSZ-X fibers. After: C/SiCON-X fibers. The SEM images were used to determine the diameter of fibers. The quantitative analysis of the average diameter and standard deviation of the mean values was carried out by ImageJ software.

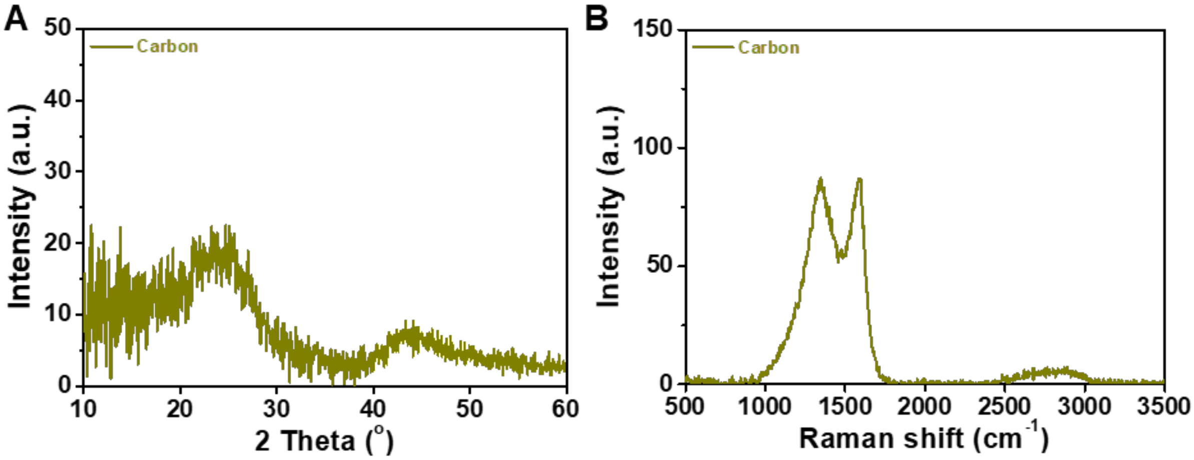

Fig. S7.

Characterization of pure carbon nonwovens. (**A**) XRD analysis of the pure carbon nonwovens. (**B**) Raman spectra of the pure carbon nonwovens.

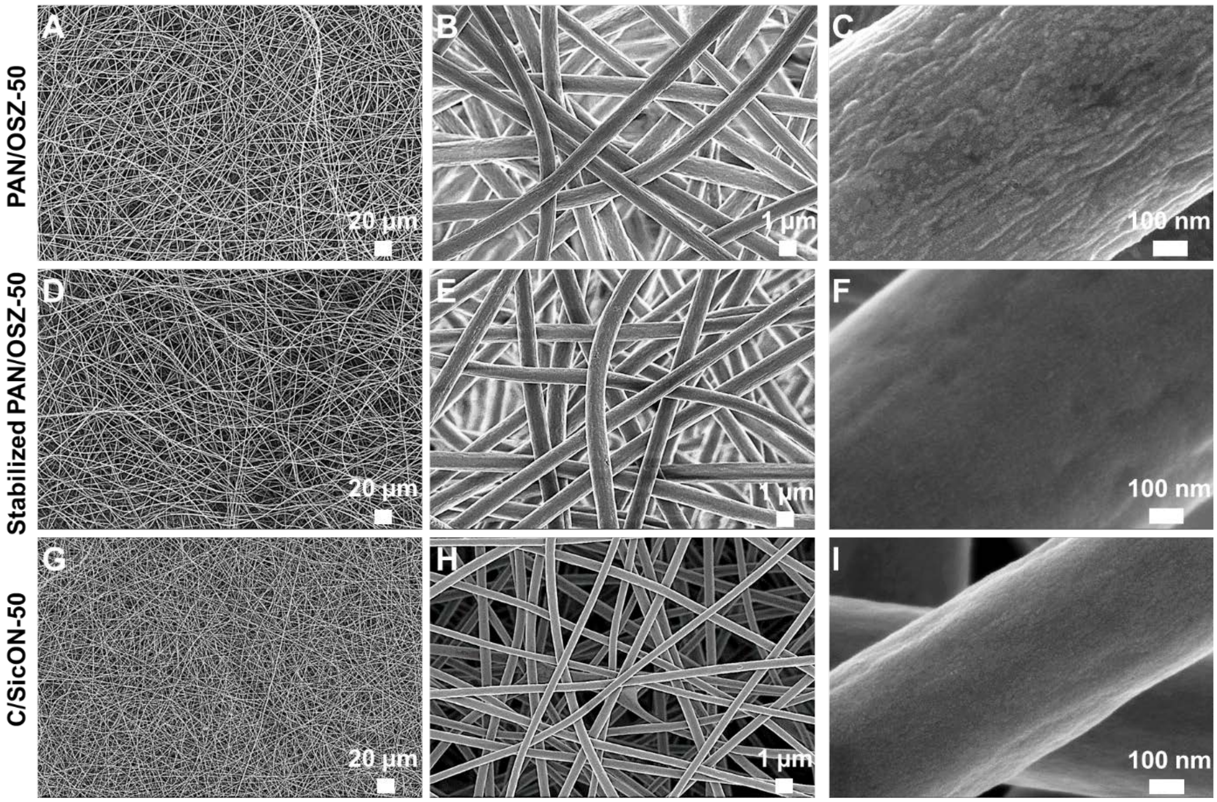

Fig. S8.

Surface SEM images of nonwovens with different magnifications. (**A-C**) PAN/OSZ-50 nonwoven. (**D-F**) stabilized PAN/OSZ-50 nonwoven. (**G-I**) C/SiCON-50 nonwoven.

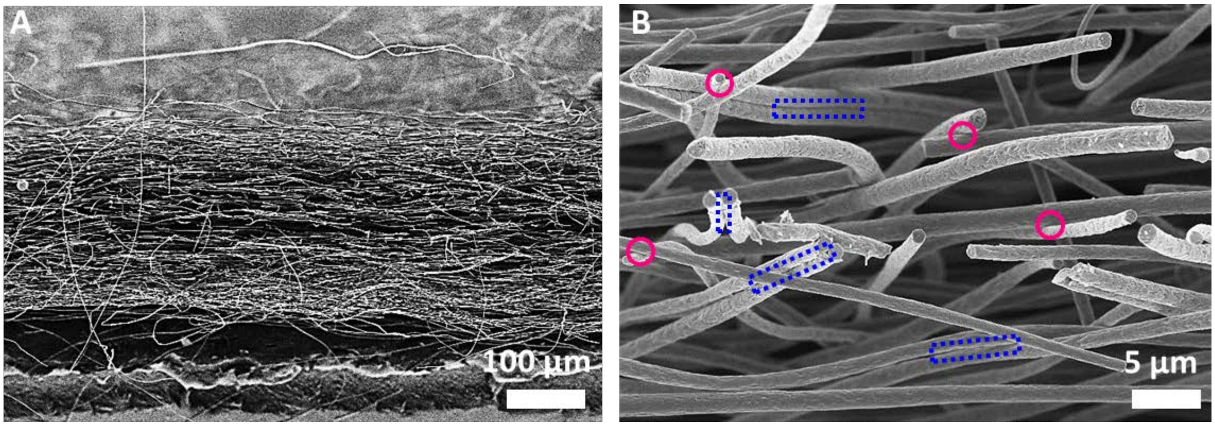

Fig. S9.

SEM images of the cross-section of C/SiCON-50 nonwoven in different magnifications. The two typical fiber-fiber contact points are marked in B: 1, pink circles (solid line) are the intersection points of two fibers; 2, blue rectangles (dotted line) are the side-by-side contacts.

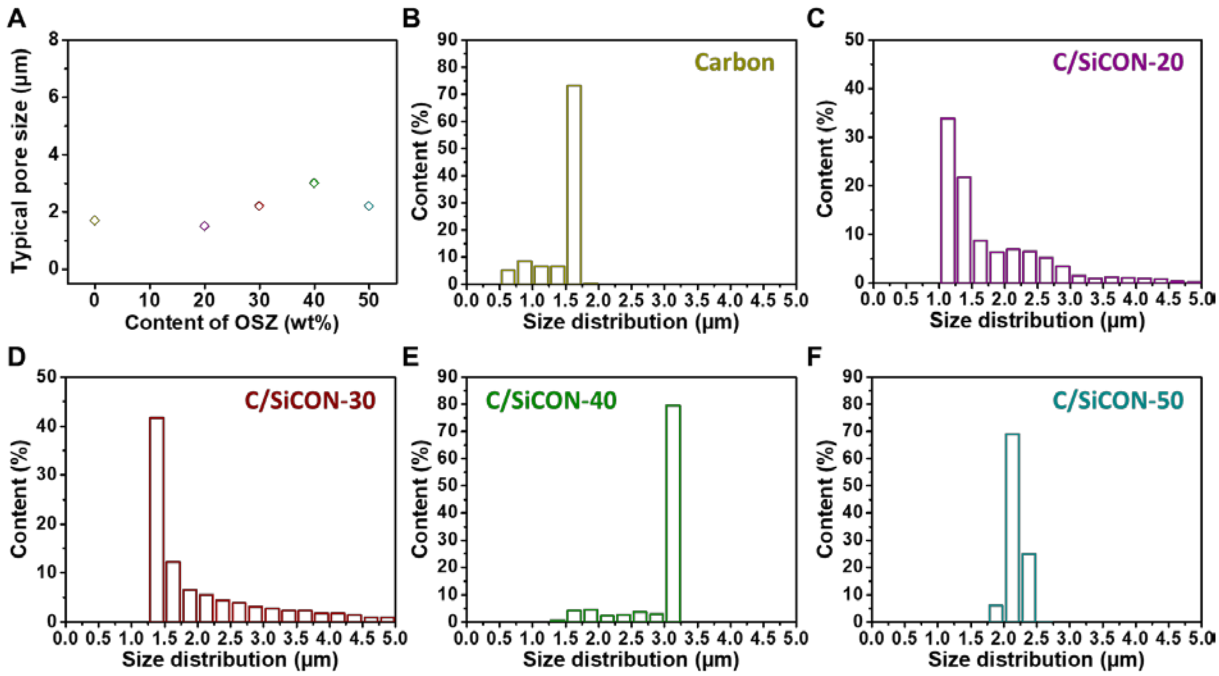

Fig. S10.

Pore size analysis of nonwovens with different contents of OSZ to PAN. (**A**) Changes in typical pore size as the function of the content of OSZ. (**B-F**) Size distribution of nonwovens.

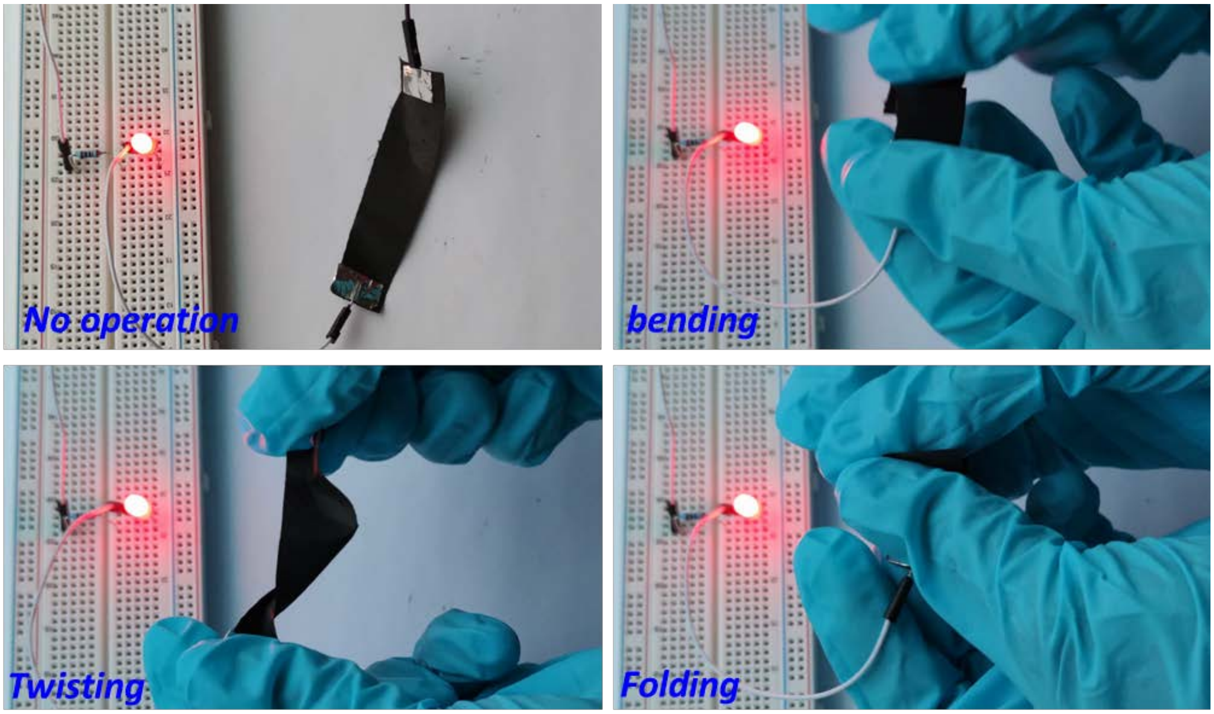

Fig. S11.

Digital photos showing the C/SiCON-50 nonwoven lighting a LED lamp under bending, twisting, and folding operations.

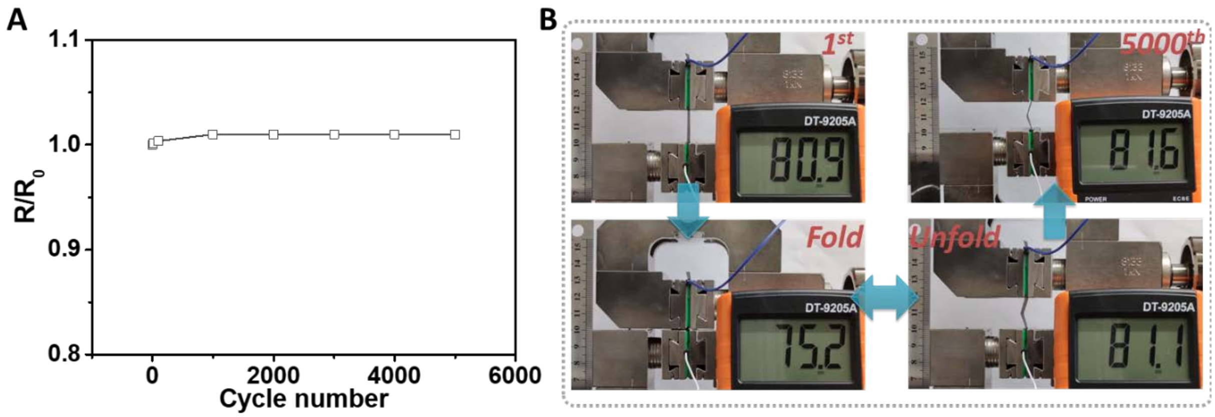

Fig. S12.

Analysis of the foldability of the nonwoven. (**A**) Resistance ratio change of C/SiCON-50 during the 5000-cycle folding-unfolding test (folding strain from 10% to 99%). (**B**) Photographs of the folding test at the 1st cycle and unfolding test at the 5000th cycle.

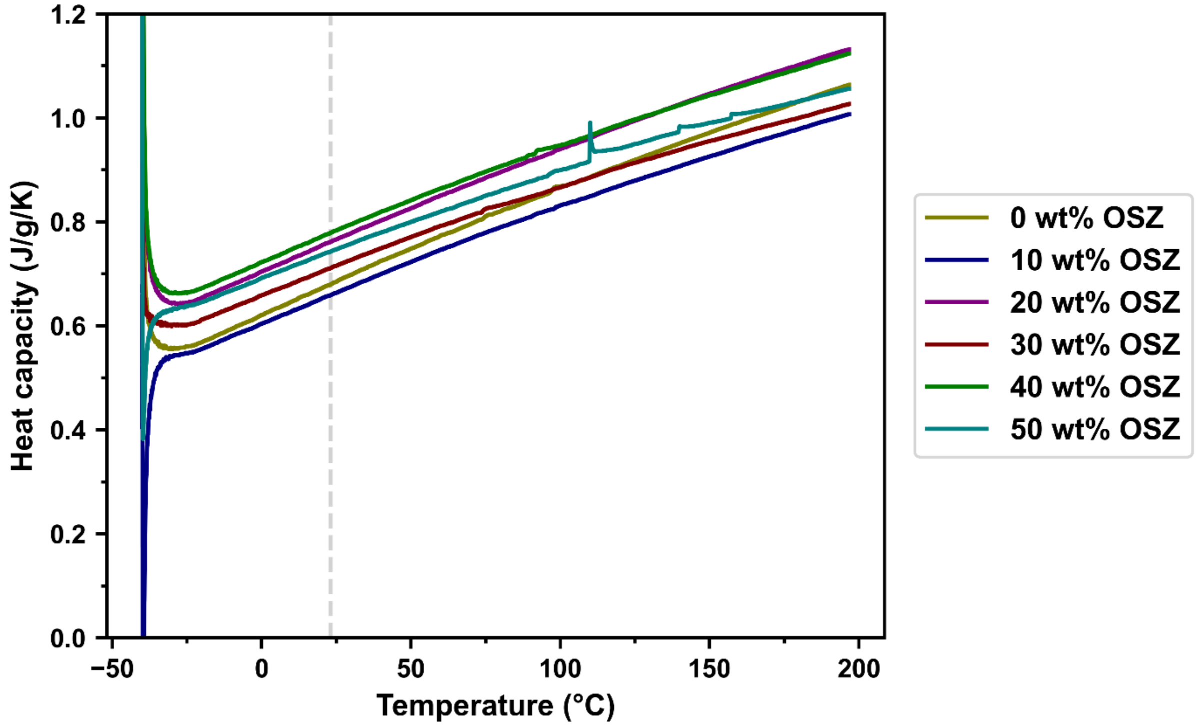

Fig. S13.

Temperature-dependent heat capacity for different OSZ contents. An increase of the heat capacity with increasing temperature can be seen. No phase transition is visible in the investigated temperature region. For evaluation of thermal conductivity, the average value at 23°C (dashed line) for four different measurements was taken.

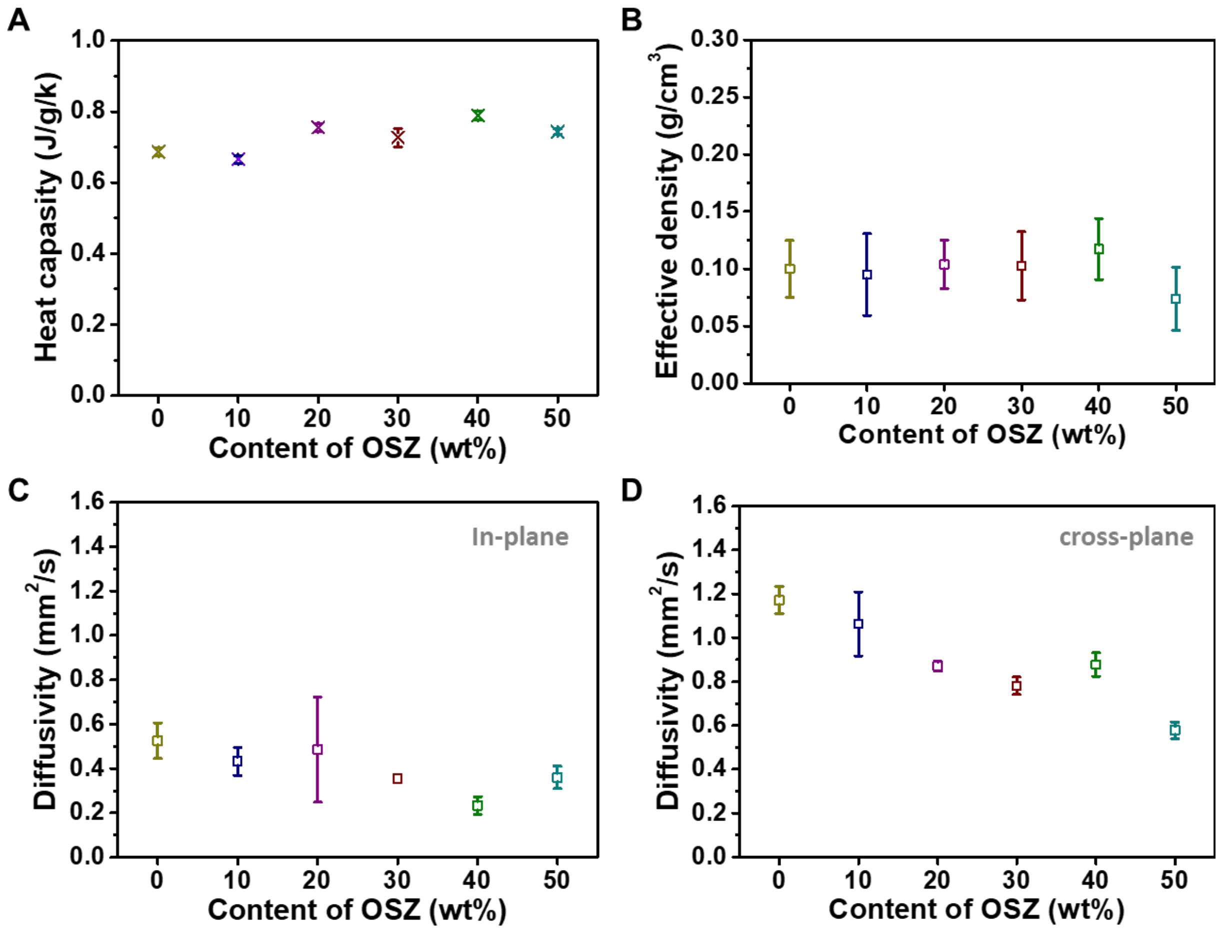

Fig. S14.

Characterization of nonwovens with different contents of OSZ to PAN. (**A**) heat capacity, (**B**) effective density, (**C**) in-plane diffusivity, and (**D**) cross-plane diffusivity.

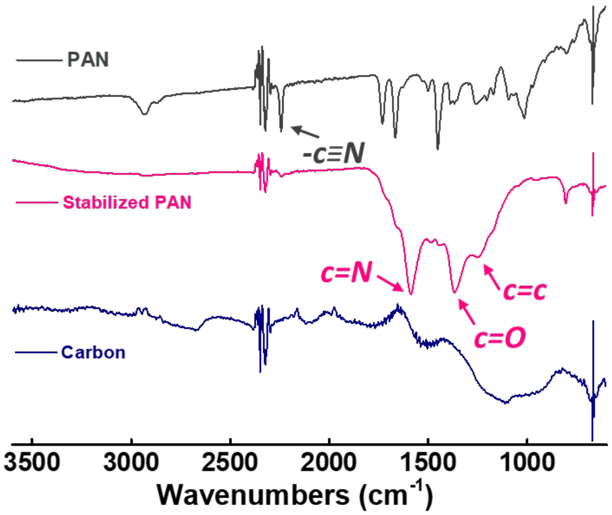

Fig. S15.

ATR-FTIR spectra of the polymer, stabilized and carbonized nonwovens from PAN.

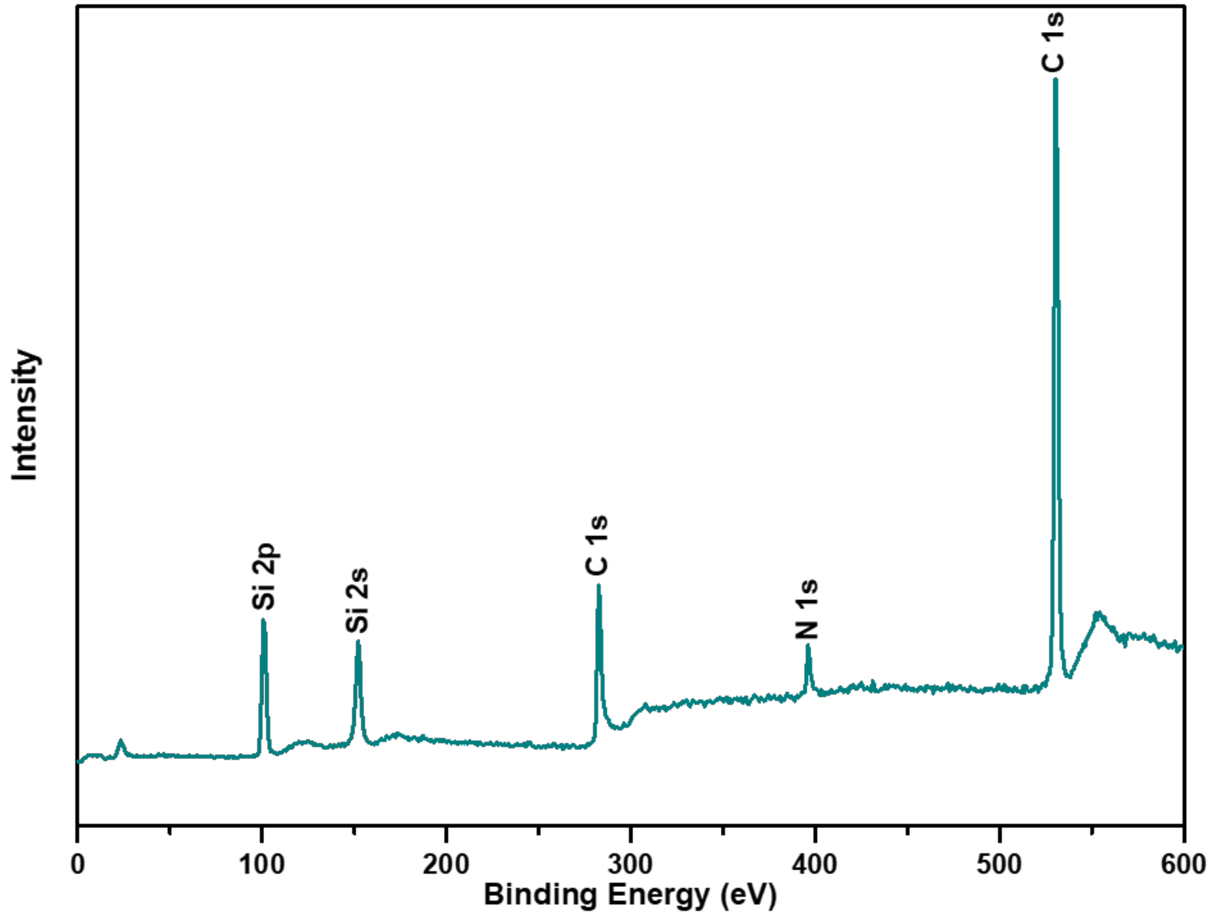

Fig. S16.

Full XPS spectra of C/SiCON-50 nonwoven. An atomic ratio of C:Si:N:O species of 31.6:17.6:5.6:5.6 % (equal to 22.5:29.5:4.7:43.5 wt%).

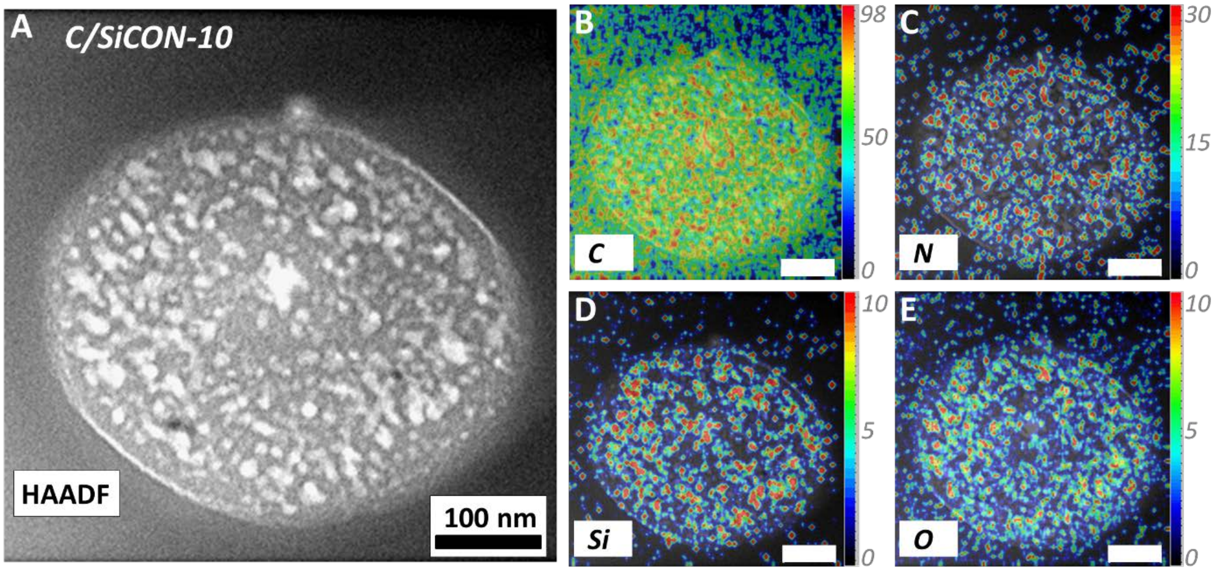

Fig. S17.

Nanostructure and elemental distribution of the C/SiCON-10 fiber. (**A**) High-resolution HAADF-STEM cross-section image. (**B-E**) EDS mapping (wt%) images showing a field of single C/SiCON-10 fiber's cross-section. Scar bars are 100 nm.

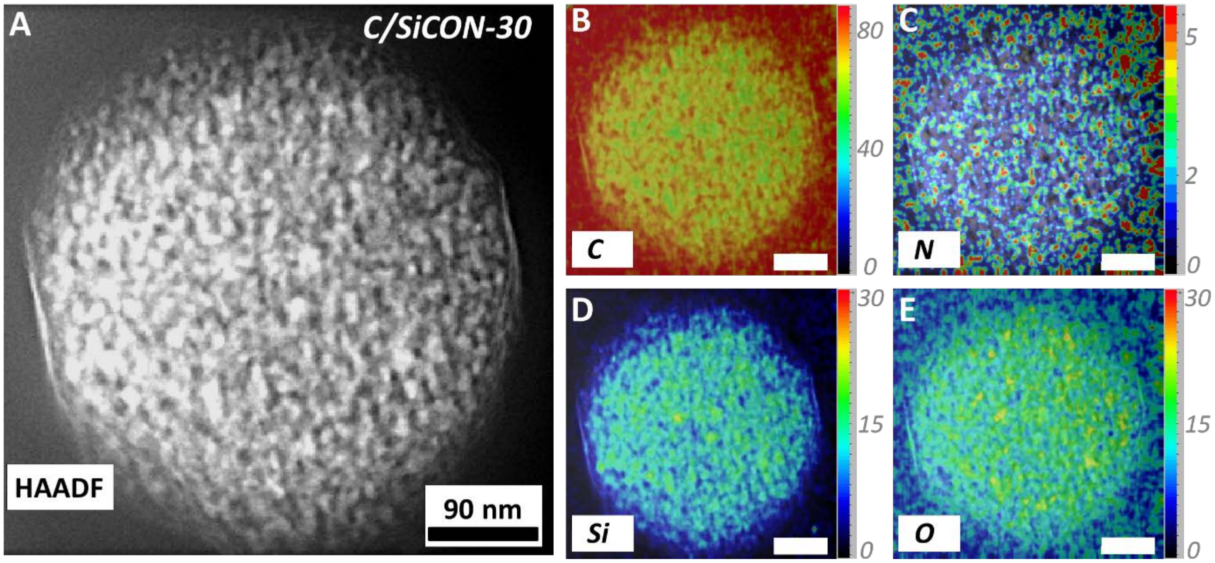

Fig. S18.

Nanostructure and elemental distribution of the C/SiCON-30 fiber. (**A**) High-resolution HAADF-STEM cross-section image. (**B-E**) EDS mapping (wt%) images showing a field of single C/SiCON-30 fiber's cross-section. Scar bars are 90 nm.

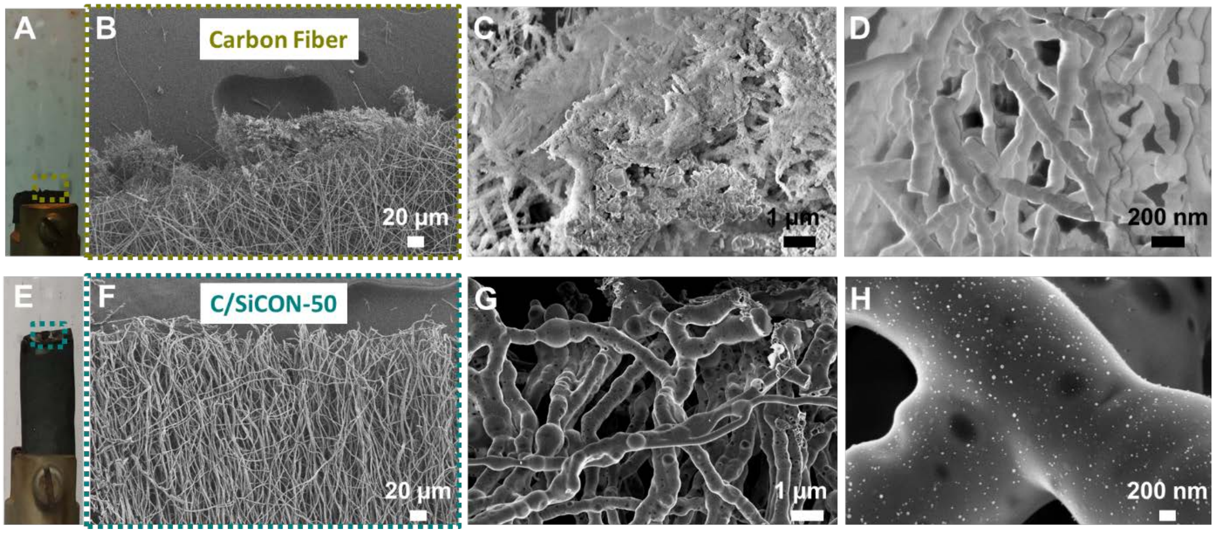

Fig. S19.

Digital photos and SEM images of nonwovens after LOI testing. (**A-D**) carbon nonwovens and (**E-H**) C/SiCON-50 nonwovens.

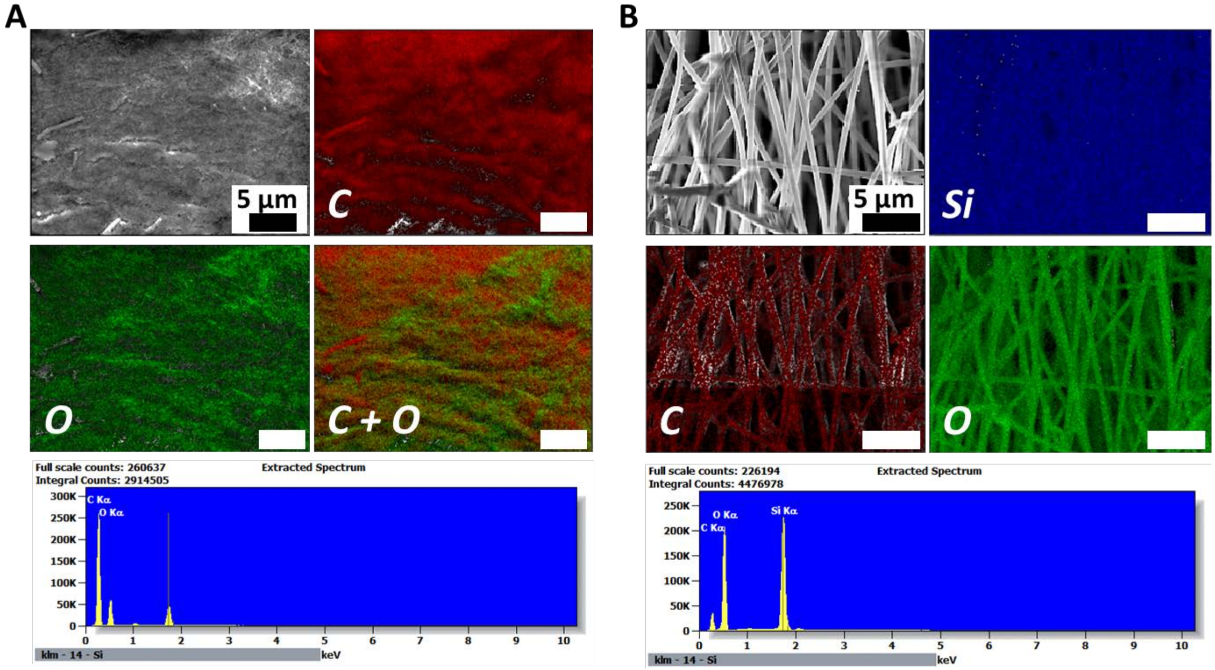

Fig. S20.

SEM and EDS maps of the nonwovens after LOI testing. (**A**) carbon nonwovens and (**B**) C/SiCON-50 nonwovens (B). The C and O weight percentages are 53.6 wt% and 46.4 wt% during the carbon nonwoven after LOI testing, respectively. The C, O, and Si weight percent are 10.4 wt%, 46.5 wt%, and 43.1 wt% during the C/SiCON-50 nonwoven after LOI testing, respectively.

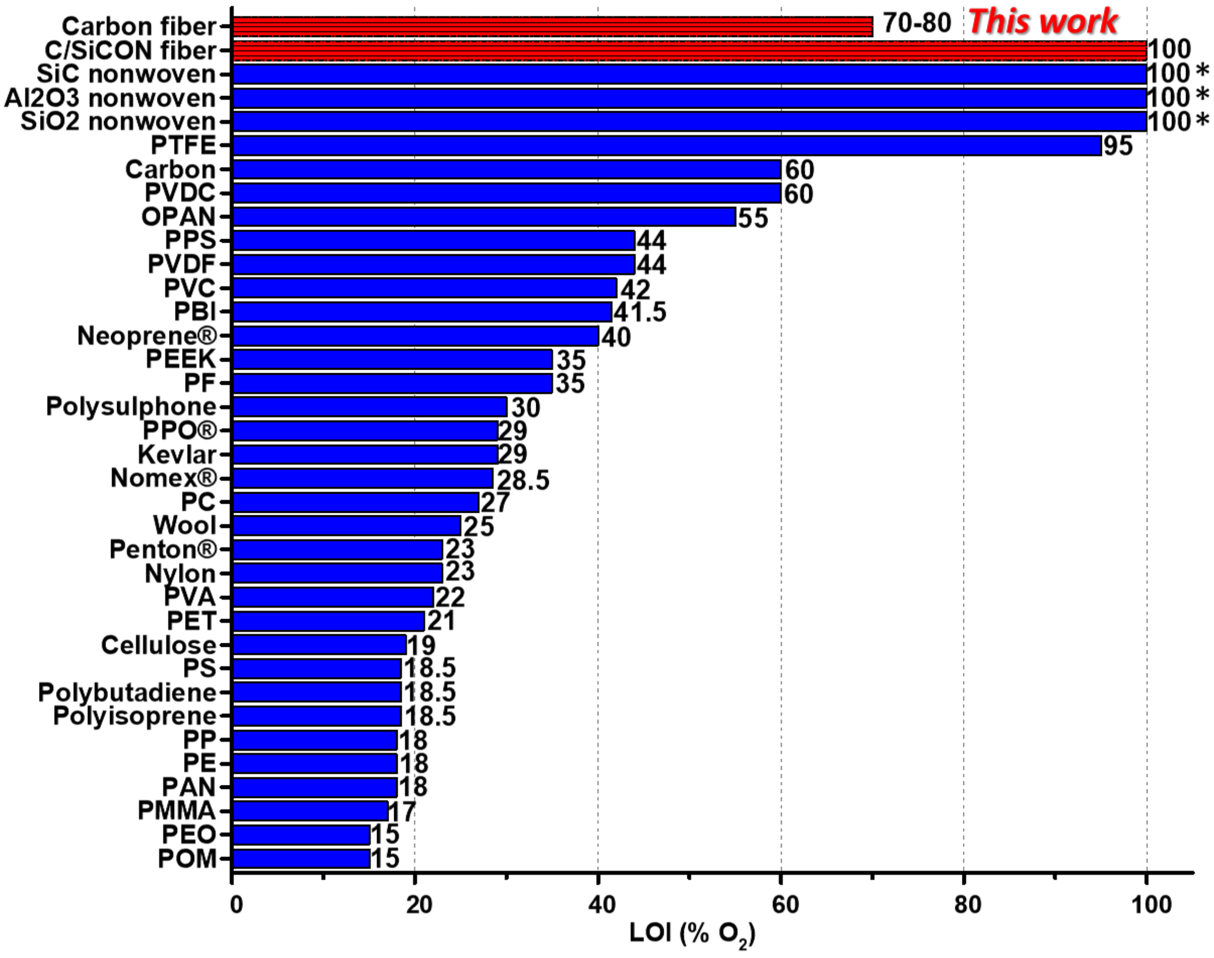

Fig. S21

Comparison of the LOI values between carbon nonwovens, C/SiCON nonwovens, and other materials (*: Measured by ourselves). The data in the plot are taken from the book: Properties of polymers, Chapter 26 - Product properties (II) Environmental behaviour and failure (*31*).

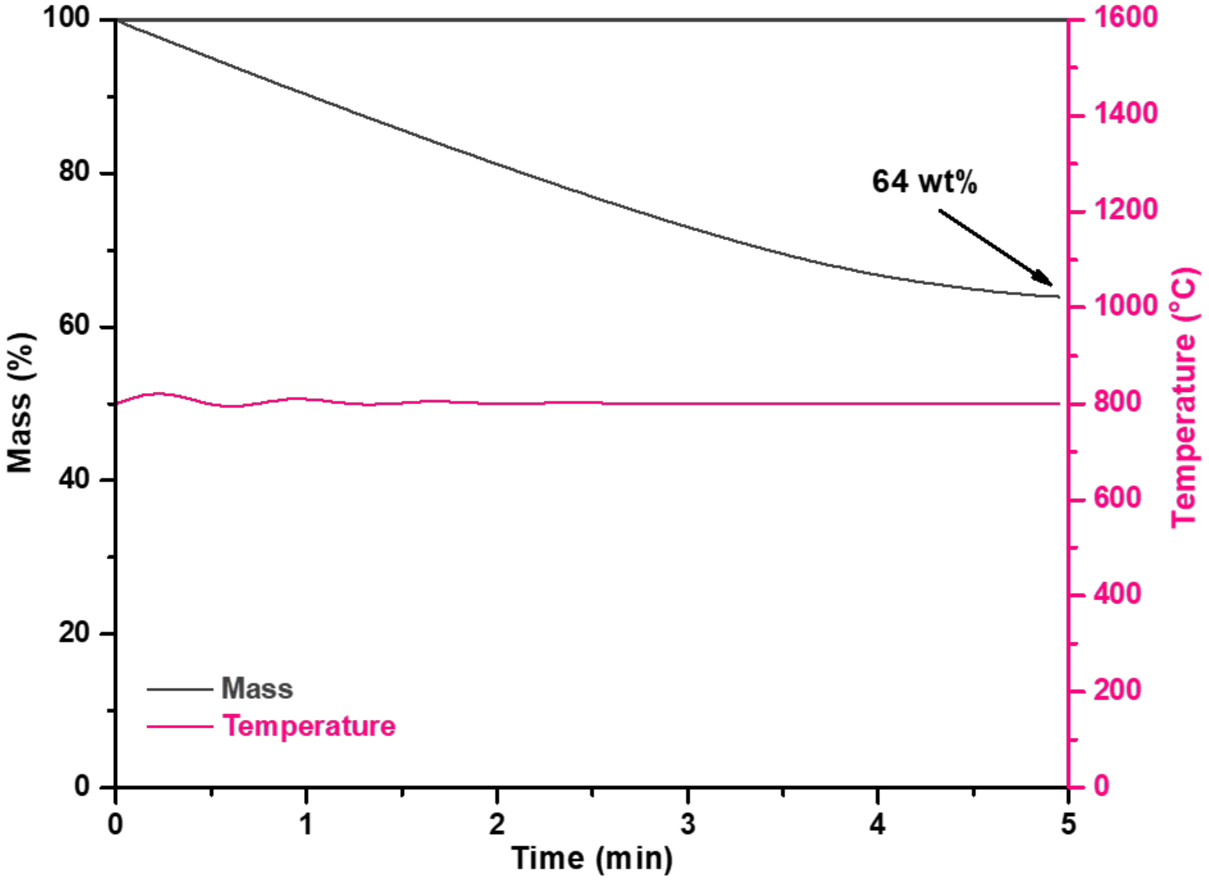

Fig. S22.
Isothermal measurement of C/SiCON-50 nonwoven at 800 °C for 5 min in air.

**Content**	**PAN**	**OSZ**	**DMF**	**Acetone**
wt%	g	g	g	g
0	1.0	0	4.7	0.96
10	1.0	0.11	5.10	0.57
20	1.0	0.25	5.53	0.61
30	1.0	0.43	6.03	0.67
40	1.0	0.67	6.03	0.67
50	1.0	1.0	6.40	0.67

Table S1.

Compositions of the electrospinning solutions. The solutions had 3 wt% DCP with respect to the weight of OSZ.

Materials	Thermal conductivity	Electrical conductivity	Density	Ref.
	mW/m/K	S/cm	mg/cm^3	
Graphene Aerogels	18	1.3	2-10	(*32*)
carbon nanofiber - polymer composites	170	$7*10^{-6}$	820-1200	(*16*)
Graphene-templated carbon aerogels	27	2.25	23.5	(*11*)
Graphene/carbon nanotube aerogels	100	0.005	31-40	(*33*)
Graphene films	$1.94*10^6$	10000	2000	(*12*)
Graphene fibers	$1.3*10^6$	2210	1450-1850	(*13*)
Carbon nanofiber nonwovens	In-plane: 1.2-15.7 ($*10^4$) Cross-plane: 428-711	--	106-739	(*15*)
Amouphous carbon	$2.6*10^4$	--	3200-3300	(*17*)
Carbon nanofiber aerogels	26.5	--	6.6	(*34*)
Nanocellulose/graphene oxide foam	15	--	7	(*35*)
Graphene aerogel	4	--	2-6	(*36*)
SiO_2/GO composite aerogel	7.2-8.9	--	80-190	(*37*)
Nitrogen-doped graphene aerogel	12.58	--	32	(*38*)

Reduced graphene oxide/polyimide aerogel	9	--	9.2	(*39*)
Graphene oxide and polyimide aerogel	12-22	--	9-16	(*40*)
Carbon aerogels	29	0.6	82	(*41*)
Carbon nanotube aerogels	16.5		4.6	(*42*)
Graphene-based foam	5.75	--	2.4	(*43*)
Hexagonal boron nitride aerogels	20-24	Insulator	0.1-10	(*20*)
Si_3N_4 nanofelts	11 (Ar)	Insulator	21	(*22*)
SiC@SiO_2 nanowire aerogel	14	Insulator	6.5	(*21*)
Silica aerogels	15.9	Insulator	180	(*19*)
**This work**	In-plane: 31.8 cross-plane: 10-19.8	4.2	100	

Table S2.

Thermal conductivity, electrical conductivity and density of materials in relevant additional literature.

	2θ (°)	FWHM (°)	d_{0002} spacing (Å)[a]	crystallite size, L_c (Stacking Height) (nm)[b]	crystallite size, L_a (Lateral Size) (nm)[c]
C-fiber	25.0	7.23	3.56	1.17	
	43.7	6.24			2.80
C/SiCON-50	25.4	8.65	3.49	0.98	
	43.8	6.65			2.63

[a]: $2d*\sin\theta = n\lambda$, $\lambda = 0.154$ nm.

[b]: $L_c = 0.94\lambda / (B(2\theta) * \cos(\theta))$, $B(2\theta)$ is FWHM.

[c]: $L_a = 1.84\lambda / (B(2\theta) * \cos(\theta))$, $B(2\theta)$ is FWHM.

Table S3.

XRD analysis of the pure carbon and C/SiCON-50 nonwovens. The information of the nanocrystallites was calculated based on the XRD analysis.

Element	PAN/OSZ-50	Stabilized PAN/OSZ-50	C/SiCON-50
	wt%	wt%	wt%
**C**	48.6	43.3	47.0
**Si**	14.0	16.1	23.2
**O**	8.6	16.3	21.8
**N**	17.4	13.1	6.5
**H**	6.0	4.18	0.5

Table S4.

Elemental analysis result of the nanofibrous nonwovens: PAN/OSZ-50, stabilized PAN/OSZ-50, and C/SiCON-. The measurements were carried out at Mikroanalytisches Labor Pascher (www.mikrolabor.com, Germany). 206

Movie S1.
Foldability of C/SiCON-50 nonwoven compared to printing paper.

Movie S2.
Flexible C/SiCON-50 nonwoven suffering the 5000-cycle folding-unfolding test with negligible electrical resistance change.

Movie S3.
Flexible C/SiCON-50 nonwoven conducting LED lamp.

Movie S4.
LOI tests of carbon nonwoven (80 % oxygen) and C/SiCON-50 nonwoven (100% oxygen).

Movie S5.
Burning test of carbon nonwoven and C/SiCON-50 nonwoven at about 800 degrees Celsius for 5 min.

REFERENCES AND NOTES

1. X. Qian, J. Zhou, G. Chen, Phonon-engineered extreme thermal conductivity materials. *Nat. Mater.* **20**, 1188–1202 (2021).

2. Q. D. Gibson, T. Zhao, L. M. Daniels, H. C. Walker, R. Daou, S. Hébert, M. Zanella, M. S. Dyer, J. B. Claridge, B. Slater, M. W. Gaultois, F. Corà, J. Alaria, M. J. Rosseinsky, Low thermal conductivity in a modular inorganic material with bonding anisotropy and mismatch. *Science* **373**, 1017–1022 (2021).

3. D. J. Voneshen, K. Refson, E. Borissenko, M. Krisch, A. Bosak, A. Piovano, E. Cemal, M. Enderle, M. J. Gutmann, M. Hoesch, M. Roger, L. Gannon, A. T. Boothroyd, S. Uthayakumar, D. G. Porter, J. P. Goff, Suppression of thermal conductivity by rattling modes in thermoelectric sodium cobaltate. *Nat. Mater.* **12**, 1028–1032 (2013).

4. J. S. Kang, H. Wu, M. Li, Y. Hu, Intrinsic low thermal conductivity and phonon renormalization due to strong anharmonicity of single-crystal Tin selenide. *Nano Lett.* **19**, 4941–4948 (2019).

5. W. Li, S. Lin, M. Weiss, Z. Chen, J. Li, Y. Xu, W. G. Zeier, Y. Pei, Crystal structure induced ultralow lattice thermal conductivity in thermoelectric Ag_9AlSe_6. *Adv. Energy Mater.* **8**, 1800030 (2018).

6. S. K. Saha, Exploring the origin of ultralow thermal conductivity in layered BiOCuSe. *Phys. Rev. B* **92**, 041202 (2015).

7. J. Yang, B. Zhang, A. D. Christodoulides, Q. Xu, A. Zangiabadi, S. R. Peurifoy, C. K. McGinn, L. Dai, E. Meirzadeh, X. Roy, M. L. Steigerwald, I. Kymissis, J. A. Malen, C. Nuckolls, Solution-processable superatomic thin-films. *J. Am. Chem. Soc.* **141**, 10967–10971 (2019).

8. B. Jiang, Y. Yu, J. Cui, X. Liu, L. Xie, J. Liao, Q. Zhang, Y. Huang, S. Ning, B. Jia, B. Zhu, S. Bai, L. Chen, S. J. Pennycook, J. He, High-entropy-stabilized chalcogenides with high thermoelectric performance. *Science* **371**, 830–834 (2021).

9. J. J. Kuo, M. Wood, T. J. Slade, M. G. Kanatzidis, G. J. Snyder, Systematic over-estimation of lattice thermal conductivity in materials with electrically-resistive grain boundaries. *Energ. Environ. Sci.* **13**, 1250–1258 (2020).

10. G. Chen, Size and interface effects on thermal conductivity of superlattices and periodic thin-film structures. *J. Heat Mass Transf.* **119**, 220–229 (1997).

11. W. Sun, A. Du, G. Gao, J. Shen, G. Wu, Graphene-templated carbon aerogels combining with ultra-high electrical conductivity and ultra-low thermal conductivity. *Microporous Mesoporous Mater.* **253**, 71–79 (2017).

12. L. Peng, Z. Xu, Z. Liu, Y. Guo, P. Li, C. Gao, Ultrahigh thermal conductive yet superflexible graphene films. *Adv. Mater.* **29**, 1700589 (2017).

13. G. Xin, T. Yao, H. Sun, S. M. Scott, D. Shao, G. Wang, J. Lian, Highly thermally conductive and mechanically strong graphene fibers. *Science* **349**, 1083–1087 (2015).

14. Y. Wang, Y. Chen, S. D. Lacey, L. Xu, H. Xie, T. Li, V. A. Danner, L. Hu, Reduced graphene oxide film with record-high conductivity and mobility. *Mater. Today* **21**, 186–192 (2018).

15. N. K. Mahanta, A. R. Abramson, M. L. Lake, D. J. Burton, J. C. Chang, H. K. Mayer, J. L. Ravine, Thermal conductivity of carbon nanofiber mats. *Carbon* **48**, 4457–4465 (2010).

16. L. C. Herrera-Ramírez, M. Cano, R. G. de Villoria, Low thermal and high electrical conductivity in hollow glass microspheres covered with carbon nanofiber-polymer composites. *Compos. Sci. Technol.* **151**, 211–218 (2017).

17. Y. Shang, Z. Liu, J. Dong, M. Yao, Z. Yang, Q. Li, C. Zhai, F. Shen, X. Hou, L. Wang, N. Zhang, W. Zhang, R. Fu, J. Ji, X. Zhang, H. Lin, Y. Fei, B. Sundqvist, W. Wang, B. Liu,Ultrahard bulk amorphous carbon from collapsed fullerene. *Nature* **599**, 599–604 (2021).

18. S.S. Roy, M.S. Arnold, Improving graphene diffusion barriers via stacking multiple layers and grain size engineering. *Adv. Funct. Mater.* **23**, 3638–3644 (2013).

19. S. Zhao, G. Siqueira, S. Drdova, D. Norris, C. Ubert, A. Bonnin, S. Galmarini, M. Ganobjak, Z. Pan, S. Brunner, G. Nyström, J. Wang, M. M. Koebel, W. J. Malfait, Additive manufacturing of silica aerogels. *Nature* **584**,387–392 (2020).

20. X. Xu, Q. Zhang, M. Hao, Y. Hu, Z. Lin, L. Peng, T. Wang, X. Ren, C. Wang, Z. Zhao, C. Wan, H. Fei, L. Wang, J. Zhu, H. Sun, W. Chen, T. Du, B. Deng, G. J. Cheng, I. Shakir, C. Dames, T. S. Fisher, X. Zhang, H. Li, Y. Huang, X. Duan, Double-negative-index ceramic aerogels for thermal superinsulation. *Science* **363**, 723–727 (2019).

21. L. Su, H. Wang, M. Niu, S. Dai, Z. Cai, B. Yang, H. Huyan, X. Pan, Anisotropic and hierarchical SiC@SiO_2 nanowire aerogel with exceptional stiffness and stability for thermal superinsulation. *Sci. Adv.* **6**, eaay6689 (2020).

22. M. Biesuz, E. Zera, M. Tomasi, P. Jana, O. Ersen, W. Baaziz, A. Lindemann, G. D. Sorarù, Polymer-derived Si_3N_4 nanofelts for flexible, high temperature, lightweight and easy-manufacturable super-thermal insulators. *Appl. Mater. Today* **20**, 100648 (2020).

23. D. P. Anderson, Carbon fiber morphology. 2. Expanded wide-angle x-ray diffraction studies of carbon fibers. Wright-Patterson, Air Force Base, Dayton, Ohio (Accession Number: ADA235599), (1991).

24. L. F. B. Ribeiro, O. Flores, P. Furtat, C. Gervais, R. Kempe, R. A. F. Machado, G. Motz, A novel PAN/silazane hybrid polymer for processing of carbon-based fibres with extraordinary oxidation resistance. *J. Mater. Chem. A* **5**, 720–729 (2017).

25. E. Zussman, X. Chen, W. Ding, L. Calabri, D. A. Dikin, J. P. Quintana, R. S. Ruoff, Mechanical and structural characterization of electrospun PAN-derived carbon nanofibers. *Carbon,* **43**, 2175–2185 (2005).

26. S. Prilutsky, E. Zussman, Y. Cohen, The effect of embedded carbon nanotubes on the morphological evolution during the carbonization of poly (acrylonitrile) nanofibers. *Nanotechnology* **19**, 165603 (2008).

27. C. A. Klein, STB model and transport properties of pyrolytic graphites. *J. Appl. Phys.* **35**, 2947–2957 (1964).

28. A. Philipp, N.W. Pech-May, B. A. F. Kopera, A. M. Lechner, S. Rosenfeldt, M. Retsch, Direct measurement of the in-plane thermal diffusivity of semitransparent thin films by lock-in thermography: An extension of the slopes method. *Anal. Chem.* **91**, 8476–8483 (2019).

29. A. Mendioroz, R. Fuente-Dacal, E. Apiñaniz, A. Salazar, Thermal diffusivity measurements of thin plates and filaments using lock-in thermography. *Rev. Sci. Instrum.* **80**, 074904 (2009).

30. O. Philips'Gloeilampenfabrieken, A method of measuring specific resistivity and Hall effect of discs of arbitrary shape. *Philips Res. Rep.* **13**, 1–9 (1958).

31. D. Van Krevelen, K. Te Nijenhuis, "[Product properties (II) Environmental behaviour and failure]" in *Properties of Polymers*, (Elsevier, 2009), chap. 26. pp. 847–873.

32. X. Xu, Q. Zhang, Y. Yu, W. Chen, H. Hu, H. Li, Naturally dried graphene aerogels with superelasticity and tunable poisson's ratio. *Adv. Mater.* **28**, 9223–9230 (2016).

33. L. C. Herrera-Ramírez, M. Cano, R. G. de Villoria, Low thermal and high electrical conductivity in hollow glass microspheres covered with carbon nanofiber-polymer composites. *Compos. Sci. Technol.* **151**, 211–218 (2017).

34. Z. Fan, D. Z. Y. Tng, C. X. T. Lim, P. Liu, S. T. Nguyen, P. Xiao, A. Marconnet, C. Y. H. Lim, H. M. Duong, Thermal and electrical properties of graphene/carbon nanotube aerogels. *Colloids Surf. A Physicochem. Eng. Asp.* **445**, 48–53 (2014).

35. N. K. Mahanta, A. R. Abramson, M. L. Lake, D. J. Burton, J. C. Chang, H. K. Mayer, J. L. Ravine, Thermal conductivity of carbon nanofiber mats. *Carbon* **48**, 4457–4465 (2010).

36. X. Hou, R. Zhang, D. Fang, Superelastic, fatigue resistant and heat insulated carbon nanofiber aerogels for piezoresistive stress sensors. *Ceram. Int.* **46**, 2122–2127 (2020).

37. B. Wicklein, A. Kocjan, G. Salazar-Alvarez, F. Carosio, G. Camino, M. Antonietti, L. Bergström, Thermally insulating and fire-retardant lightweight anisotropic foams based on nanocellulose and graphene oxide. *Nat. Nanotechnol.* **10**, 277–283 (2015).

38. Y. Xie, S. Xu, Z. Xu, H. Wu, C. Deng, X. Wang, Interface-mediated extremely low thermal conductivity of graphene aerogel. *Carbon* **98**, 381–390 (2016).

39. Y. Lei, Z. Hu, B. Cao, X. Chen, H. Song, Enhancements of thermal insulation and mechanical property of silica aerogel monoliths by mixing graphene oxide. *Mater. Chem. Phys.* **187**, 183–190 (2017).

40. C. Yue, J. Feng, J. Feng, Y. Jiang, Efficient gaseous thermal insulation aerogels from 2-dimension nitrogen-doped graphene sheets. *Int. J. Heat Mass Transf.* **109**, 1026–1030 (2017).

41. Q. Peng, Y. Qin, X. Zhao, X. Sun, Q. Chen, F. Xu, Z. Lin, Y. Yuan, Y. Li, J. Li, W. Yin, C. Gao, F. Zhang, X. He, Y. Li, Superlight, mechanically flexible, thermally superinsulating, and antifrosting anisotropic nanocomposite foam based on hierarchical graphene oxide assembly. *ACS Appl. Mater. Interfaces* **9**, 44010–44017 (2017).

42. Y. Qin, Q. Peng, Y. Zhu, X. Zhao, Z. Lin, X. He, Y. Li, Lightweight, mechanically flexible and thermally superinsulating rGO/polyimide nanocomposite foam with an anisotropic microstructure. *Nanoscale Adv.* **1**, 4895–4903 (2019).

43. X. Lu, O. Nilsson, J. Fricke, R. W. Pekala, Thermal and electrical conductivity of monolithic carbon aerogels. *J. Appl. Phys.* **73**, 581–584 (1993).

44. Y. W. Chen, H. Zhan, J. N. Wang, A direct foaming approach for carbon nanotube aerogels with ultra-low thermal conductivity and high mechanical stability. *Nanoscale* **13**, 11878–11886 (2021).

45. M. J. Oh, J. H. Lee, P. J. Yoo, Graphene-based ultralight compartmentalized isotropic foams with an extremely low thermal conductivity of 5.75 mW m^{-1} K^{-1}. *Adv. Funct. Mater.* **31**, 2007392 (2020).

46. X. Xu, Q. Zhang, M. Hao, Y. Hu, Z. Lin, L. Peng, T. Wang, X. Ren, C. Wang, Z. Zhao, C. Wan, H. Fei, L. Wang, J. Zhu, H. Sun, W. Chen, T. Du, B. Deng, G. J. Cheng, I. Shakir, C. Dames, T. S. Fisher, X. Zhang, H. Li, Y. Huang, X. Duan, Double-negative-index ceramic aerogels for thermal superinsulation. *Science* **363**, 723–727 (2019).

47. M. Biesuz, E. Zera, M. Tomasi, P. Jana, O. Ersen, W. Baaziz, A. Lindemann, G. D. Sorarù, Polymer-derived Si_3N_4 nanofelts for flexible, high temperature, lightweight and easy-manufacturable super-thermal insulators. *Appl. Mater. Today* **20**, 100648 (2020).

48. L. Su, H. Wang, M. Niu, S. Dai, Z. Cai, B. Yang, H. Huyan, X. Pan, Anisotropic and hierarchical SiC@ SiO_2 nanowire aerogel with exceptional stiffness and stability for thermal superinsulation. *Sci. Adv.* **6**, eaay6689 (2020).

49. S. Zhao, G. Siqueira, S. Drdova, D. Norris, C. Ubert, A. Bonnin, S. Galmarini, M. Ganobjak, Z. Pan, S. Brunner, G. Nyström, J. Wang, M. M. Koebel, W. J. Malfait, Additive manufacturing of silica aerogels. *Nature* **584**, 387–392 (2020).
